# Bioactive Constituents of *F. esculentum* Bee Pollen and Quantitative Analysis of Samples Collected from Seven Areas by HPLC

**DOI:** 10.3390/molecules24152705

**Published:** 2019-07-25

**Authors:** Feng Li, Sen Guo, Shanshan Zhang, Sainan Peng, Wei Cao, Chi-Tang Ho, Naisheng Bai

**Affiliations:** 1 College of Chemical Engineering, Department of Pharmaceutical Engineering, Northwest University, Taibai North Road 229, Xi’an 710069, China; 2 College of Food Science and Technology, Northwest University, Taibai North Road 229, Xi’an 710069, China; 3 Department of Food Science, Rutgers University, 65 Dudley Road, New Brunswick, NJ 08901, USA

**Keywords:** bee pollen, enzyme inhibitory activity, HPLC-DAD, GC-MS, quality analysis

## Abstract

Bee pollen contains all the essential amino acids needed by humans. China is the largest producer of bee pollen in the world. In the present study, we identified 11 fatty acids in *F. esculentum* bee pollen oil by GC-MS analysis, and 16 compounds were isolated from *F. esculentum* bee pollen by column chromatography and identified. A high-performance liquid chromatography-diode array detector (HPLC-DAD) method was established for the quality control of *F. esculentum* bee pollen. A validated HPLC-DAD method was successfully applied to the simultaneous characterization and quantification of nine main constituents in seven samples collected from seven different areas in China. The results showed that all standard calibration curves exhibited good linearity (*R*^2^ > 0.999) in HPLC-DAD analysis with excellent precision, repeatability and stability. The total amount in the samples from the seven regions ranged from 23.50 to 46.05 mg/g. In addition, seven compounds were studied for their bioactivity using enzymic methods, whereby kaempferol (**3**) showed high α-glucosidase inhibitory activity (IC_50_: 80.35 μg/mL), ergosterol peroxide (**8**) showed high tyrosinase inhibitory activity (IC_50_: 202.37 μg/mL), and luteolin (**1**) had strong acetylcholinesterase inhibitory activity (IC_50_: 476.25 μg/mL). All results indicated that *F. esculentum* bee pollen could be a nutritious health food.

## 1. Introduction

Bee pollen (BP), one of the hive products in addition to honey, royal jelly and propolis, is gaining attention due to the presence of bioactive compounds associated with beneficial health properties [1,2]. The BP composition varies due to the plant species and is also influenced by age, nutritional condition of the plant, differences in gathering area and time as well as environmental conditions during pollen development [3,4,5,6]. Monofloral pollen pellets maintain organoleptic and biochemical properties similar to those of the original plant, whereas the multifloral pollen has variable properties of more than two original plants [7]. Generic bee pollen composition data were considered sufficient for most purposes, but now the usefulness of bee pollen-specific composition data is increasingly being acknowledged [8,9]. Recent research has also shown that bee pollen possesses therapeutic benefits for improving the cardiovascular system, stimulating body immunity [10], promoting antitumor effects, delaying aging [11], scavenging free radicals, regulating intestinal function [12], and treating prostate disease [13,14,15,16,17]. 

α-Glucosidase (AG) is an important enzyme to maintain the normal metabolism of the human body. It hydrolyzes maltose and sucrose by catalyzing the hydrolysis of 1,4-glycosidic bonds in sugar, which is widely distributed in human body and plays a crucial role in human metabolism [18]. Acetylcholinesterase (AChE) is a major functional enzyme that hydrolyzes acetylcholine (ACh) in the central nervous system. Anatomical studies have found that AChE protein expression in the brain of Alzheimer’s disease (AD) patients is elevated [19,20]. In recent years, there have been more and more studies on acetylcholinesterase inhibitors [21]. The drugs approved by the US Food and Drug Administration (FDA) for the treatment of AD are mainly AChE inhibitors, including donepezil, rivastigmine and tacrine [22], but they also have significant side effects and toxicity, and low bioavailability. Therefore, the search for new natural products as a safe and non-toxic AChE inhibitors is urgently needed.

Melanin is one of the natural pigments responsible for skin pigmentation and hair color in mammals [23]. The excessive production of melanin results in hyperpigmentation dysfunctions, along with aging [24]. Tyrosinase, a copper-containing enzyme involved in melanin biosynthesis [25], is the only enzyme found to regulate the generation of melanin in humans. The investigation of tyrosinase inhibitors may lead to the development of new skin-whitening substances, medicinal agents and anti-browning constituents [26]. Various potential tyrosinase inhibitors from natural products have been found, and they have attracted attention because of their safety and abundance [27]. This promote us to investigate the tyrosinase inhibitory activity isolated from *F. esculentum* bee pollen.

In the light of this, we studied the composition of *F. esculentum* pollens and quantitatively analyzed *F. esculentum* pollens collected from different areas in China. Nine compounds, including flavonoids, phenolic acids and polyphenols were analyzed by a validated HPLC-DAD method. We also carried out enzyme activity experiments to verify their α-glucosidase inhibitory activity, tyrosinase inhibitory activity and acetylcholinesterase inhibitory activity. The obtained quantitative data will be useful for quality control of *F. esculentum* pollens for their comprehensive use as a functional food.

## 2. Materials and Methods

### 2.1. Chemicals, Regents and Materials

Seven batches of BPs, consisting of *Fagopyrum esculentum Moench* from different production areas in China, were collected in 2016. These samples were identified by the corresponding author (NB). Their voucher specimens were deposited in a refrigerator at 4 °C at the Department of Pharmaceutical Engineering, Northwest University, China. BPSX was from Shannxi, BPAH was from Anhui, BPNM was from Neimeng, BPHN was from Heinan, BPGS was from Gansu, BPHB1 was from Hebei and BPHB2 was from Hubei.

**Figure 1:**
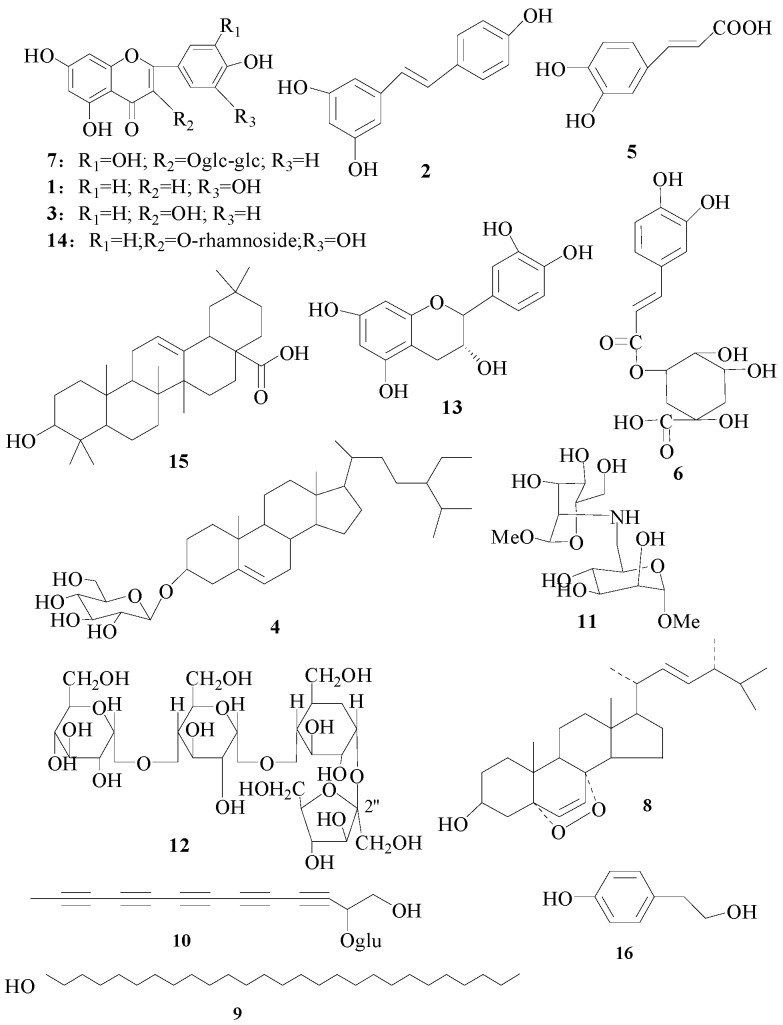
Chemical structures of the 16 identified compounds in *F. esculentum* pollens.

Silica gel (100–200 mesh), MCI GEL CHP-20P (Mitsubishi Kasei Co., Tokyo, Japan) and Sephadex LH-20 (Sigma Chemical Co., St. Louis, MO, USA) were used for column chromatographic separations. Thin layer chromatography (TLC) was performed on Sigma-Aldrich silica gel TLC plates (250 μm thickness, 2–25 μm particle size). Acetonitrile was of HPLC grade from Merck (Darmstadt, Germany). Water was purified with a sub boil high purity water still (SYZ 550, Tianjin, China). Other reagents such as ethyl alcohol, CH_2_Cl_2_ and methanol used in this experiment were of analytical grade and purchased from Hengxing Chemical Reagent Co., Ltd. (Tianjin, China). The 4-nitrophenyl β-d-glucopyranoside (PNPG), acarbose, l-arginine, kojic acid, acetylthiocholine iodide (ACTI), 5,5′-dithiobis(2-nitrobenzoic acid) (DTNB), and huperzine A used in the activity experiments were all from Shanghai Aladdin Biochemical Technology Co., Ltd. (Shanghai, China). We have isolated the standard substances **1**–**16** and their structures were fully characterized by chemical and spectroscopic methods (NMR and MS data are provided as Supplementary Materials). They are luteolin (**1**), resveratrol (**2**), kaempferol (**3**), β-daucosterol (**4**), caffeic acid (**5**), chlorogenic acid (**6**), rutin (**7**), ergosterol peroxide (**8**), octacosanol (**9**), 2-β-d-glucopyranosyloxy-1-hydroxytrideca-3,5,7,9,11-pentayne (**10**), α-d-Alt-*p*-OMe (N2-6) α-d-Gle-*p*-OMe (**11**), *O*-α-d-glucopyranosyl-(1→4)-α-d-glucopyranosyl-(1→4)-α-d-glucopyranosyl-(1→2)-β-d-fructofuranoside (**12**), catechin (**13**), quercitrin (**14**), oleanolic acid (**15**) and tyrosol (**16**). The purity of each compound was >98%, determined by HPLC analysis. The chemical structures of these reference compounds are shown in Figure 1.

### 2.2. HPLC Conditions

HPLC analysis was performed on a 1260 LC Series system (Agilent, Palo Alto, CA, USA) using a Luna C-18 column (5 micron, 4.6 mm I.D. × 250, Phenomenex, Inc. Torrance, CA, USA) with a flow rate of 1.0 mL/min, and the column temperature was maintained at 20 °C. Injection volume were 10 µL for the standard solutions and 15 µL for sample solutions. The mobile phase was composed of C (methanol) and D (water) with a gradient elution: 0 min, 95% D; 0–6 min, 95–92% D; 6–11 min, 92–90% D; 11–17 min, 90–86% D; 17–25 min, 86–75% D; 25–30 min, 75–70% D; 30–40 min, 70–45% D; 40–47 min, 45–30% D; 47–55 min, 30–5% D; 55–70 min, 5–95% D. At the end of the run, 95% of acetonitrile was used to flush the column for 5 min and an additional 5 min of post run time were set to allow for equilibration of the column with the starting eluant. UV detector was set at 210 nm wavelength for the compounds during the experiment.

### 2.3. Saponification and Methylation for Fatty Acids (FAs)

The *F. esculentum* pollen oils were obtained by a pressing method under low temperature, and processed by saponification and methylation reactions for further GC-MS analysis. Briefly, 0.5 g of *F. esculentum* pollens oil prepared above was dissolved in 4 mL methanol solution containing 0.5 mol/L of potassium hydroxide, stirred for 2 min, and kept in a 60 °C water bath for 1 h in a capped test tube to induce saponification for derivatization. Then, 3 mL of methanol solution containing 10% concentrated sulphuric acid was added, the sample was again placed in a 60 °C water bath for 1 h. After reactions, the sample was cooled to room temperature. Subsequently, 3 mL of deionized water and 6 mL of n-hexane were added for extraction. After centrifugation (13,000 rpm, 10 min), the supernatant was obtained and dried using a nitrogen flow. The obtained solution was stored in a refrigerator at 4 °C and filtered through a 0.45 μm nylon-membrane filter prior to injection into the GC-MS analysis.

### 2.4. GC-MS Analysis 

The FA profiles in the pollen oils were analyzed by GC-MS as their corresponding methyl ester. The oil sample was analyzed three times under the same conditions. The GC-MS (Agilent 6590*N* Network System, M 5973*N*) instrument was coupled with a Rtx-5MS column (30 m × 0.25 mm × 0.25 μm (5% phenylmethylsiloxane)). Helium (purity, 99.99%) was used as the carrier gas at a flow rate of 3.0 mL/min. The injection volume is 1 μL by split (1:30). Injector, source and oven temperatures were 250 °C, 200 °C and 120 °C, respectively. The initial oven temperature 120 °C held for 2.5 min and then ramped at 10 °C/min to 180 °C, ramped at 1.5 °C/min to 210 °C, ramped at 5 °C/min to 250 °C and last for 3 min.

### 2.5. Extraction and Isolation

The BPs (15 kg from Shanxi) was percolated with 90% EtOH at room temperature for three times, then the EtOH extracts were combined. The extract (6.32 kg) was suspended in H_2_O (6.5 L) and then extracted exhaustively with PE, EtOAc and *n*-BuOH (6.5 L each) 3 times. The *n*-BuOH solution containing 195.3 g of solid was chromatographed on a silica gel column (15 L, 8.0 cm × 120 cm) with CH_2_Cl_2_-MeOH (100:1–0:1) gradient to give seven crude fractions (Fr.1–7).

Fr.2, Fr.3 and Fr.4 were repeatedly subjected to silica gel column with CH_2_Cl_2_-EtOAc, then they were repeatedly subjected to MCI GEL CHP-20P column with water-MeOH system (start from H_2_O and increased MeOH ratio to 10–100% MeOH) to give compound **7** (21 mg from fraction 2-3-3, *t*_R_ = 48.34 min), **16** (21 mg from fraction 3-3, *t*_R_ = 51.04 min), **3** (12 mg from fraction 4-3, *t*_R_ = 49.10 min), **5** (10 mg from fraction 4-2-2, *t*_R_ = 30.47 min) and **6** (9 mg from fraction 4-3, *t*_R_ = 29.53 min). Fr. 5 and Fr. 6 were repeatedly subjected to MCI GEL CHP-20P column and/or Sephadex LH-20 column with water-MeOH system (start from H_2_O and increased MeOH ratio to 10–100% MeOH) to give compound **13** (25 mg from fraction 5-4, *t*_R_ = 44.85 min), **14** (26 mg from fraction 5-3, *t*_R_ = 54.91 min), **1** (15 mg from fraction 6-2-2, *t*_R_ = 47.89 min), **2** (16 mg from fraction 6-4, *t*_R_ = 32.26 min) and **9** (38 mg from fraction 6-2-3, *t*_R_ = 62.25 min). The separation process of other compounds is also as described above. Compound **4** (18 mg from fraction 2-3-3, *t*_R_ = 57.82 min), **8** (35 mg from fraction 5-4, *t*_R_ = 44.01 min), **9** (38 mg from fraction 6-2-3, *t*_R_ = 62.04 min), **10** (28 mg from fraction 3-2, *t*_R_ = 31.16 min), **11** (14 mg from fraction 3-3, *t*_R_ = 36.17 min), **12** (27 mg from fraction 5-2, *t*_R_ = 53.58 min) and **15** (18 mg from fraction 4-2-2, *t*_R_ = 60.54 min). Their structures are shown in Figure 1.

### 2.6. Quantitative Analysis

#### 2.6.1. Preparation of Sample Solutions

Each BP material was ground to powder and sifted through an 80 mesh sieve. Subsequently, 5 g of pulverized samples were accurately weighed, and extracted with ultrasonication using 40 mL of 90% methanol (volume fraction) for 1.5 h. After centrifugation (13,000 rpm, 10 min), the supernatant was concentrated and transferred into a 5 mL volumetric flask. The obtained solution was stored in a refrigerator at 4 °C and filtered through a 0.45 μm filter prior to HPLC analysis.

#### 2.6.2. Preparation of Standard Solutions

Nine reference standards were accurately weighed (10,000 mg) respectively, then dissolved in methanol and a small amount of dimethyl sulfoxide to final concentrations of 1.0 mg/mL. Every standard stock solution was diluted with methanol to five appropriate concentrations (500 μg/mL, 250 μg/mL, 100 mg/mL, 50 μg/mL and 10 μg/mL). All the standard solutions were stored at 4 °C in darkness for HPLC analysis.

#### 2.6.3. Identification and Quantification

**Figure 2:**
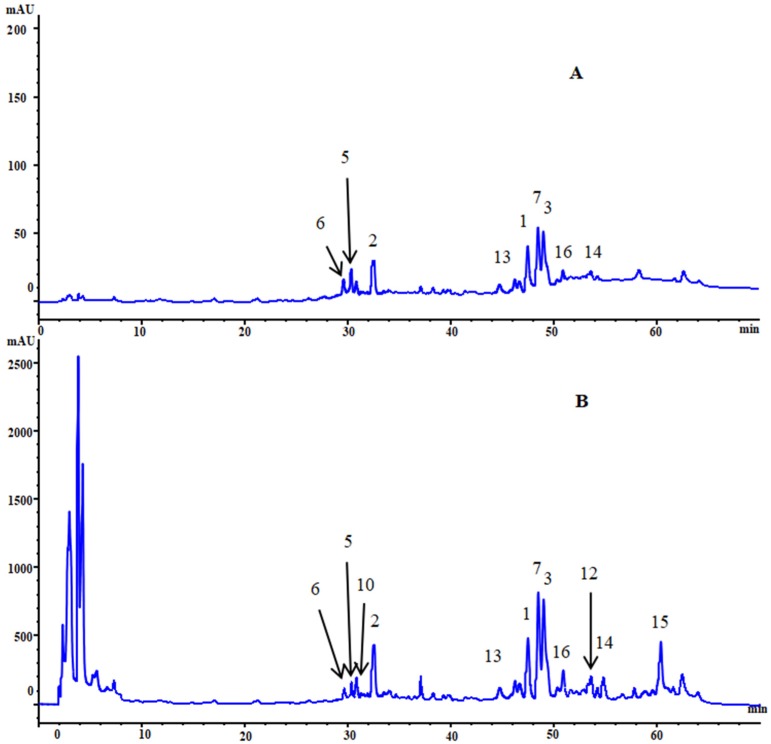
HPLC chromatograms of solution of standards (**A**) and samples (**B**) at 210 nm. Peaks: luteolin (**1**), resveratrol (**2**), kaempferol (**3**), caffeic acid (**5**), chlorogenic acid (**6**), rutin (**7**), 2-β-d-glucopyranosyloxy-1-hydroxytrideca-3,5,7,9,11-pentayne (**10**), O-α-d-glucopyranosyl-(1→4)-α-d-glucopyranosyl-(1→4)-α-d-glucopyranosyl-(1→2)-β-d-fructofuranoside (**12**), catechin (**13**), quercitrin (**14**), oleanolic acid (**15**) and tyrosol (**16**).

Identification of these analytes were carried out by comparing the HPLC retention time and UV spectra of target peaks with those of the standards. Quantification was performed on the basis of linear calibration plots of the peak areas versus the concentration. The results are presented in Figure 2. Quantification was performed on the basis of linear calibration curves. 

### 2.7. Bioactivity Assay

#### 2.7.1. α-Glucosidase Inhibitory Activity Experiment

In a 96-well plate, group A was added 20 μL of 0.1 mol/L PBS (phosphate buffered saline (pH 7.4)), 20 μL of the sample (standard solution dissolved in DMSO to the concentration of 1.0 mg/mL) and 20 μL of α-glucosidase as a sample group (*n* = 3); Group B was added 40 μL of 0.1 mol/L PBS and 20 μL of the sample as the control group (*n* = 3); Group C was added 40 μL 0.1 mol/L PBS and 20 μL α-glucosidase as blank group (*n* = 3); after incubation for 15 min at 37 °C, then 2.5 mmol/L PNPG glycoside was added to each well and incubated at 37 °C. The reaction was stopped by adding 80 μL of a 0.2 mol/L Na_2_CO_3_ solution to each well after 15 min. The absorbance at a wavelength of 405 nm was measured with a microplate reader. Acarbose was used as a positive control. The specific grouping is shown in Table 1.

#### 2.7.2. Tyrosinase Inhibitory Activity Experiment

In a 96-well plate, group A was added 80 μL of 0.1 mol/L PBS, 50 μL of solvent (solvent used to dissolve the sample: DMSO) and 50 μL of tyrosinase as a blank group (*n* = 2); group B was added 130 μL of 0.1 mol/L PBS and 50 μL of solvent as blank background group (*n* = 2); group C was added with 80 μL of 0.1 mol/L PBS, 50 μL of the sample (standard solution dissolved in DMSO to the concentration of 1.0 mg/mL) and 50 μL of tyrosinase as the experimental group (*n* = 2); group D were added 130 μL of 0.1 mol/L PBS and 50 μL of the sample as experimental background group (*n* = 2); finally, 20 μL of substrate (l-tyrosine) was added to each well to trigger the reaction, and the 96-well plate was further incubated at 37 °C. After 30 min, the absorbance at a wavelength of 405 nm was measured by a microplate reader. Kojic acid was used as a positive control. The specific grouping is shown in Table 2.

#### 2.7.3. Acetylcholinesterase Inhibitory Activity Experiment

In a 96-well plate, group A was added 140 μL of 0.1 mol/L PBS, 20 μL of the sample (standard solution dissolved in DMSO to the concentration of 1.0 mg/mL) and 20 μL of acetylcholinesterase as a sample group (*n* = 3); Group B was added with 160 μL of 0.1 mol/L PBS and 20 μL of the sample as a control group (*n* = 3); Group C was added with 160 μL 0.1 mol/L PBS and 20 μL acetylcholinesterase as a blank group (*n* = 3), the all were incubated at 4 °C for 20 min, then 10 μL of 15 mM ACTI and 10 μL of 2 mM DTNB were added per well, continue incubated at 37 °C for 20 min, the absorbance at a wavelength of 412 nm was measured with a microplate reader. Huperzine A was used as a positive control. The specific grouping is shown in Table 3.

## 3. Results and Discussion Sections in Wrong Order—Experimental is Last—Renumber Anything Affected 

### 3.1. GC-MS Analysis

**Figure 3:**
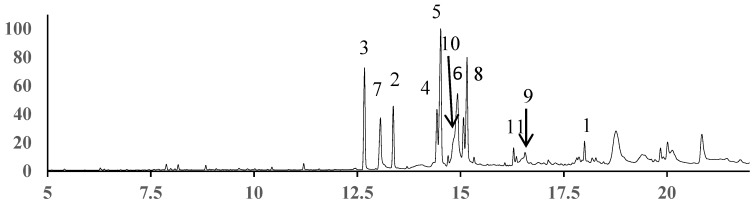
GC chromatograms of solution of samples. Peaks: 1, Tetradecanoic acid, methyl ester; 2, Hexadecanoic acid, methyl ester; 3, Hexadecanoic acid, 2-methyl-, methyl ester; 4, (9*Z*,12*Z*)-octadecadienoic acid, methyl ester; 5, (9*Z*,12*Z*,15*Z*)-octadecatrienoic acid, methyl ester; 6, Octadecanoic acid, methyl ester; 7, Heptadecanoic acid, methyl ester; 8, 18-Methylnonadecanoate, methyl ester; 9, *cis*-13-Eicosenoic acid, methyl ester; 10, Eicosanoic acid, methyl ester; 11, Docosanoic acid, methyl ester.

The methylated *F. esculentum* pollen oil was subjected to GC-MS analysis in triplicate. The unambiguous identification of the separated components was carried out by comparing their recorded mass spectra with a database (the NIST08 libraries). The components in *F. esculentum* pollen oil were listed according to their elution order on the non-polar Rtx-5MS column as shown in Table 2. As shown in Figure 3, the GC-MS analysis of *F. esculentum* pollens oil sample led to identification of 11 constituents, which correspond to 85.20% of the total contents. In the present work, (9*Z*,12*Z*,15*Z*)-octadecatrienoic acid α-linolenic acid, ALA) (C18:3^Δ9,12,15^) was found to be dominant at the high level of 36.25%, which is higher than previously reported in the literature (35.58%) [28], followed by hexadecanoic acid (C16:0, 7.67%) and (9*Z*,12*Z*)-octadecadienoic acid (9,12-linoleic acid, LA) (C18:2^Δ9,12^, 3.8%) (see Table 4). ALA and LA are the predominant ω-3 and ω-6 FAs for humans. Dietary intake of ω-3 FAs is an effective prophylactic means associated with blood lipid, inflammatory, autoimmune and cardiovascular diseases [29,30,31,32]. Furthermore, other FAs including *cis*-13-eicosenoic acid (C20:1^Δ13^), heptadecanoic acid (C17:0), eicosanoic acid (C20:0), octadecanoic acid (C18:0), tetradecanoic acid (C14:0) and docosanoic acid (C22:0) were also found. 

### 3.2. Optimization of Extraction Method

**Figure 4:**
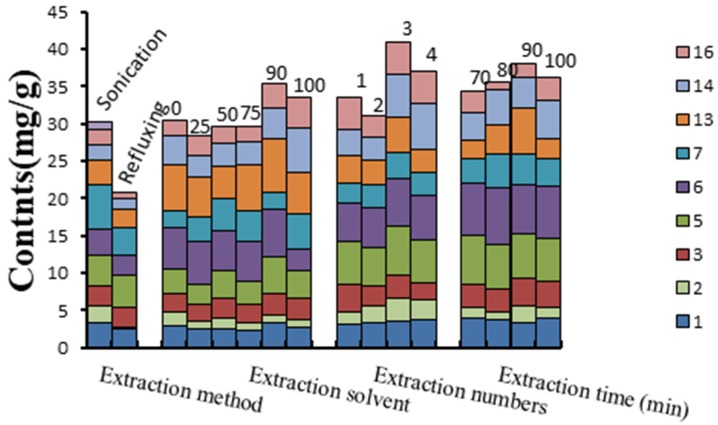
Effects of extraction method, solvent, number and time on the extraction efficiency of investigated standards in the sample (BPSX) of *F. esculentum* pollen. Note: luteolin (1), resveratrol (2), kaempferol (3), caffeic acid (5), chlorogenic acid (6), rutin (7), catechin (13), quercitrin (14), and tyrosol (16).

To determine the optimal extraction conditions and enhance the overall response of those investigated compounds in the HPLC analysis, extraction methods (refluxing and sonication), extraction solvents (100%, 90%, 75%, 50% and 25% methanol-water), number of extractions (1, 2, 3 and 4 times), and extraction times (30, 60, 90 and 120 min) were investigated individually by using a univariate approach (default values: extraction method, sonication; extraction solvent, 50% methanol; extraction number, 2 times; extraction time, 90 min) on the sample BPSX. The results suggested that the established extracted method (each sample was extracted three times with 50% methanol under ultrasonication condition for 90 min) was optimal for HPLC analysis. The results are shown in Figure 4.

### 3.3. HPLC Method Validation

The HPLC method was validated by determining the linearity, limit of detection (LOD), limit of quantitation (LOQ), precision (inter-day and intra-day), repeatability, stability, and accuracy. 

#### 3.3.1. Linearity, LODs and LOQs

Linear calibration curves were established by plotting the peak area (*Y*) versus the corresponding concentration (*X*, μg/mL) of each compound. All calibration curves exhibited good linear regressions (R^2^ ≥ 0.999) within the tested concentration ranges (Table 5). The lowest concentration of working solution for calibration use was diluted to a series of appropriate concentrations. They were then measured and checked until the signal-to-noise ratio (S/*N*) for each compound was approximately 3 for LOD and 10 for LOQ. The LODs and LOQs of all analytes were less than 0.23 and 0.64 µg/mL, respectively. 

#### 3.3.2. Precision, Repeatability, and Stability

As all nine analytes could be detected and quantified in BPSX, BPSX was selected for the tests of precision, repeatability, and stability. The intra-day and inter-day precisions were successively evaluated by a prepared sample solution under optimal conditions within one day and duplicating this analysis once a day for three consecutive days, respectively. A stability test of the sample solution was conducted at 0, 2, 4, 8, 12, 24 and 48 h. Repeatability was determined by analyzing six independently prepared solutions of sample BPSX. The relative standard deviation (RSD) of peak area for each marker compound was taken as a measure (Table 6). The RSD values of the reference compounds were found in the ranges of 0.71–1.45%, 0.95–2.25%, 0.47–2.06%, and 1.60–3.15% for intra-day variations, inter-day variations, stability, and repeatability.

#### 3.3.3. Recovery

To check the accuracy of the analytical method, a recovery test was performed. In the test, a known amount of standards was added into 0.1 g of sample BPSX previously quantified. The spiked samples were extracted, processed and quantified per the methods above. Three replicates were performed for the test. The recovery values ranged from 97.25 to 102.07%, and RSDs values were less than 3.49%. These results indicate that the established method is accurate enough for quantitative analysis.

### 3.4. Quantitative Determination of Nine Compounds

The proposed analytical method was then successfully applied to the simultaneous quantification of seven BPSs collected from different areas in China. The results (Table 7) indicated that the contents of nine compounds varied greatly among different samples. The total content of these investigated compounds reached as high as 46.05 mg/g in sample BPNM, which was cultivated in Neimeng. However, the content was only 23.50 mg/g in BPGS, for the sample cultivated from Gansu. Flavonoids were considered the most abundant constituents in BPSs. Rutin, an important bioflavonoid, is abundantly found in various foodstuffs [17]. Rutin has been acknowledged for its protective and beneficial effects on various aspects of the biological system, rutin possesses sufficient potential for increasing immune activity by cellular and humoral mediated mechanisms [33]. Analyte **1** (luteolin) and analyte **2** (resveratrol) were detected in seven samples, the contents of analyte **1** varied from 6.23 to 10.94 mg/g and analyte **2** varied from 3.26 to 5.25 mg/g. In addition, compound **6** could not be detected in many analyzed samples, only be detected in BPSX and BPNM, and the contents were 1.45 and 1.12 mg/g, respectively.

Chlorogenic acid and caffeic acid are common bioactive compounds [34,35]. Chlorogenic acid has the effect of protecting liver, inhibits cancer cell growth and has a beneficial effect on ameliorating aging-related diseases [24]. Caffeic acid has also been reported have to a wide antibacterial effect [25]. From our data, analyte **6** (chlorogenic acid) varied from 1.12 to 1.45 mg/g, and analyte **5** (caffeic acid) varied from 3.47 to 6.00 mg/g. Look at all the data, the highest contents of analyte **1** is 10.94 mg/g in sample BPNM, the lowest contents of analyte **7** (rutin) is 1.05 mg/g in sample BPHB1. 

### 3.5. Activity Analysis

#### 3.5.1. Analysis of α-Glucosidase Inhibitory Activity

Glucosidase is an important member of the sugar metabolism pathway in vivo, and α-glucosidase is directly involved in the metabolic pathways of starch and glycogen [36]. By inhibiting α-glucosidase, the chemical metabolism of sugar can be reduced, thereby achieving a hypoglycemic effect [37]. 

From Table 8, compound **3** (kaempferol), **7** (rutin), **8** (ergosterol peroxide), **10** (2-β-d-glucopyranosyloxy-1-hydroxytrideca-3,5,7,9,11-pentayne), **11** (α-d-Alt-*p*-OMe (N2-6) α-d-Gle-*p*-OMe) and **12** (O-α-d-glucopyranosyl-(1→4)-α-d-glucopyranosyl-(1→4)-α-D-glucopyranosyl-(1→2)-β-d-fructofuranoside) had significant α-glucosidase inhibitory activity compared with the positive control group, the IC_50_ value of the acarbose as the positive control group, which was 515.98 μg/mL, and the IC_50_ value of the compound **3** was the lowest, which was 80.35 μg/mL, and the IC_50_ value of the compound **7** was 188.42 μg/mL, indicating that *F. esculentum* bee pollen has hypoglycemic effects. Notably, compounds **8**, **10**, **11** and **12** were tested for α-glucosidase inhibitory activity for the first time, and the difference in their IC_50_ value was not significant. The inhibition mechanism of kaempferol on α-glucosidase activity has been investigated by multispectroscopic methods and kaempferol had a significant inhibitory activity on α-glucosidase with an IC_50_ value of (1.16 ± 0.04) × 10^−5^ mol L^−1^ and *K*_i_ value of (1.31 ± 0.03) × 10^−5^ mol L^−1^ [38].

#### 3.5.2. Analysis of Tyrosinase Inhibitory Activity

From Table 9, compounds **1** (luteolin), **8** (ergosterol peroxide), **11** (α-d-Alt-*p*-OMe (N2-6) α-d-Glep-OMe), and **12** (O-α-d-glucopyranosyl-(1→4)-α-d-glucopyranosyl-(1→4)-α-d-glucopyranosyl-(1→2)-β-d-fructofuranoside) had significant tyrosinase inhibitory activity compared with the positive control group, the IC_50_ value of the kojic acid as the positive control group, which was 517.07 μg/mL, indicating that *F. esculentum* bee pollen has hypoglycemic effect. The IC_50_ value of the compound **8** was the lowest, which was 202.37 μg/mL, followed by the compound **8**, which was 302.42 μg/mL, compared with the positive control group, their IC_50_ value were low. The phenolic extracts from rape bee pollen have been shown to have anti-tyrosinase activity, which could reduce the content of melanin and inhibit the production of melanin in B16 mouse melanoma cells through the cAMP/MITF/TYR pathway [39], and the evaluation of the biological activity of isolated compounds from bee pollen of *Quercus mongolica* revealed that the polyamine derivatives with coumaroyl and caffeoyl moieties showed tyrosinase inhibition with IC_50_ values of 19.5–85.8 μM [40].

#### 3.5.3. Analysis of Acetylcholinesterase Inhibitory Activity

From Table 10, compound **1** (luteolin), **3** (kaempferol), **7** (rutin), **8** (ergosterol peroxide), **10** (2-β-d-glucopyranosyloxy-1-hydroxytrideca-3,5,7,9,11-pentayne), **11** (α-d-Alt-*p*-OMe (N2-6) α-d-Gle-*p*-OMe), and **12** (*O*-α-d-glucopyranosyl-(1→4)-α-d-glucopyranosyl-(1→4)-α-d-glucopyranosyl-(1→2)-β-d-fructofuranoside) has strong acetylcholinesterase inhibitory activity, the difference between the positive group was not significant. IC_50_ value of the huperzine A as the positive control group, which was 502.98 μg/mL, compound **10** was the highest, with IC_50_ values of 521.14 μg/mL, compound **1** was the lowest, with IC_50_ values of 476.25 μg/mL. At present, there are few studies on the inhibitory activity of acetylcholinesterase in bee pollen. Acetylcholinesterase is involved in the development and maturation of cells and can promote the development of neurons and nerve regeneration [41].

## 4. Conclusions

In this study, 16 compounds were isolated from *F. esculentum* bee pollen, including flavone compounds, phenolic acids, terpenoids, and 11 fatty acids were analyzed in *F. esculentum* bee pollen oil. Saturated fatty acids were 30.86%, unsaturated fatty acids were 69.14%, of which (9*Z*,2*Z*,15*Z*)-octadecatrienoic acid content was as high as 36.25%, indicating that the unsaturated fatty acid content of *F. esculentum* bee pollen is high, and it is a green and healthy food. A HPLC method with high stability, good repeatability and high precision was established, and the content of nine compounds in *F. esculentum* bee pollen from seven different habitats was quantitatively analyzed. From the results, the content of bee pollen collected in Neimeng was the highest (46.05 mg/g), and the content of bee pollen collected in Gansu was the lowest (23.50 mg/g). Kaempferol (**3**) showed high α-glucosidase inhibitory activity (IC_50_: 80.35 μg/mL), ergosterol peroxide (**8**) showed high tyrosinase inhibitory activity (IC_50_: 202.37 μg/mL), and luteolin (**1**) had strongly acetylcholinesterase inhibitory activity (IC_50_: 476.25 μg/mL). Furthermore, bee pollen has been used as a health food supplement for many years due to its abundant nutrient properties including proteins, polysaccharide, and lipids [42]. The average protein content of bee pollen is 24.65% (10–40% dry weight) [9]. The proportion of amino acids in bee pollen is also high and there are many kinds of amino acids. Studies have proved that the protein in bee pollen accounts for 29.18% of its dry weight [43]. Polysaccharides, namely carbohydrates, are the most abundant component in bee pollen, accounting for 18.9–57.6% of bee pollen [44]. Lipids are an important component of bee pollen, and their content accounts for about 1–20% of its dry weight [45]. Other trace elements including vitamins, minerals, enzymes, nucleic acids have also been found in pollen. Therefore, it has antioxidant, anti-inflammatory and immune-enhancing capabilities [46,47,48,49]. Our research has proved that isolated metabolites possess potential anti-aging activity. These compounds could work synergistically with nutrients in pollen and provide more evidence for the beneficial effects of pollen. In conclusion, *F. esculentum* bee pollen is a kind of natural nutrition and health food for anti-aging, beauty and improving human immunity. 

At present, most of reports on bee pollen are aimed at the study of extracts. Therefore, the innovation of this paper is the separation and identification of extracts. The quantitative analyses of bee pollen from seven different places of origin were also carried out. 

## Figures and Tables

**Table 1:** Specific grouping and reaction system for α-glucosidase inhibitory activity.

Group	Reaction System	Absorbance
A	20 μL PBS, 20 μL sample, 20 μL α-glucosidase	OD_A_
B	40 μL PBS, 20 μL sample	OD_B_
C	40 μL PBS, 20 μL α-glucosidase	OD_C_

**Table 2:** Specific grouping and reaction system for tyrosinase inhibitory activity.

Group	Reaction System	Absorbance
A	80 μL PBS, 50 μL solvent, 50 μL tyrosinase, 20 μL substrate	OD_A_
B	130 μL PBS, 50 μL solvent, 20 μL substrate	OD_B_
C	80 μL PBS, 50 μL tyrosinase, 50 μL sample, 20 μL substrate	OD_C_
D	130 μL PBS, 50 μL sample, 20 μL substrate	OD_D_

**Table 3:** Specific grouping and reaction system for acetylcholinesterase inhibitory activity.

Group	Reaction System	Absorbance
A	140 μL PBS, 20 μL sample, 20 μL acetylcholinesterase	OD_A_
B	160 μL PBS, 20 μL sample	OD_B_
C	160 μL PBS, 20 μL acetylcholinesterase	OD_C_

**Table 4:** FAs profile of the pollen oil by GC-MS analysis.

No.	Components ^a^	Content (%) ^b^	Molecular Formula
1	Tetradecanoic acid, methyl ester	2.95	C_15_H_30_O_2_
2	Hexadecanoic acid, methyl ester	7.67	C_17_H_34_O_2_
3	Hexadecanoic acid, 2-methyl-, methyl ester	25.66	C_18_H_36_O_2_
4	(9*Z*,12*Z*)-Octadecadienoic acid, methyl ester	3.8	C_19_H_34_O_2_
5	(9*Z*,12*Z*,15*Z*)-Octadecatrienoic acid, methyl ester	36.25	C_19_H_32_O_2_
6	Octadecanoic acid, methyl ester	9.36	C_19_H_38_O_2_
7	Heptadecanoic acid, methyl ester	4.57	C_18_H_36_O_2_
8	18-Methylnonadecanoate, methyl ester	3.73	C_21_H_42_O_2_
9	*cis*-13-Eicosenoic acid, methyl ester	1.39	C_21_H_40_O_2_
10	Eicosanoic acid, methyl ester	2.04	C_21_H_42_O
11	Docosanoic acid, methyl ester	2.58	C_23_H_46_O_2_

^a^ expressed the FAs or corresponding methyl esters; ^b^ denoted the average normalized peak area percent.

**Table 5:** Calibration curve data for nine reference compounds (*n* = 3).

Analytes *	Regression Equation	R^2^	Linear Range (μg/mL)	LOD (μg/mL)	LOQ (μg/mL)
1	*Y* = 83.34*X* − 446.99	R^2^=0.99929	4.00–600	0.15	0.48
2	*Y* = 62.22*X* − 249.29	R^2^ = 0.99973	1.19–250	0.07	0.37
3	*Y* = 19.68*X* − 105.92	R^2^ = 0.99918	1.50–250	0.09	0.52
5	*Y* = 14.77*X* − 79.30	R^2^ = 0.99919	2.50–250	0.11	0.63
6	*Y* = 54.15*X* − 295.55	R^2^ = 0.99915	1.00–200	0.08	0.45
7	*Y* = 9.86*X* − 52.98	R^2^ = 0.99914	3.00–250	0.14	0.39
13	*Y* = 63.91*X* − 351.28	R^2^ = 0.99912	1.50–500	0.23	0.51
14	*Y* = 49.21*X* − 271.64	R^2^ = 0.99916	2.01–800	0.15	0.46
16	*Y* = 64.13*X* − 352.54	R^2^ = 0.99902	1.08–500	0.18	0.64

* 1: luteolin, 2: resveratrol, 3: kaempferol, 5: caffeic acid, 6: chlorogenic acid, 7: rutin, 13: catechin, 14: quercitrin, 16: tyrosol.

**Table 6:** Precision, repeatability, stability, and recovery of the analytes.

Analytes	Precision (*n* = 5)		Repeatability (*n* = 6) RSD (%)	Stability (*n* = 6) RSD (%)	Recovery (*n* = 3)	
	Intra-Day RSD (%)	Inter-Day RSD (%)			Mean (%)	RSD (%)
1	0.82	1.39	2.41	1.74	97.56	2.51
2	1.23	1.24	2.05	0.94	98.69	1.99
3	0.95	2.08	3.15	1.13	97.25	2.32
5	1.17	2.25	2.53	2.04	99.64	3.49
6	1.45	1.50	1.98	0.84	98.80	3.16
7	1.11	1.61	2.47	0.98	100.56	2.98
13	0.98	1.22	2.26	1.85	99.05	2.04
14	0.71	0.95	1.60	0.47	101.12	1.63
16	0.89	1.57	1.84	2.06	98.56	1.95

1: luteolin, 2: resveratrol, 3: kaempferol, 5: caffeic acid, 6: chlorogenic acid, 7: rutin, 13: catechin, 14: quercitrin, 16: tyrosol.

**Table 7:** Contents of nine compounds in seven BPSs.

Contents ^a^ of 9 Compounds (mg/g)										
	1	2	3	5	6	7	13	14	16	Total
BPSX	9.46	5.25	3.67	3.47	1.45	1.45	2.40	2.66	2.28	32.09
BPAH	8.43	4.23	3.32	4.19	ND ^b^	1.34	9.38	4.91	1.66	37.46
BPNM	10.94	4.81	3.49	5.54	1.12	1.36	10.05	2.19	6.55	46.05
BPHN	8.68	5.03	2.59	6.00	ND	ND	2.47	5.19	3.27	33.23
BPGS	8.67	5.19	ND	5.57	ND	ND	4.07	ND	ND	23.50
BPHB1	8.54	3.26	ND	5.02	ND	1.05	3.01	ND	3.28	24.16
BPHB2	6.23	4.85	1.83	4.08	ND	ND	1.26	1.29	4.21	23.75

1: luteolin, 2: resveratrol, 3: kaempferol, 5: caffeic acid, 6: chlorogenic acid, 7: rutin, 13: catechin, 14: quercitrin, 16: tyrosol. ${}^{a}\;\mathrm{Content}=\frac{X\frac{V_{1}\left(\mathrm{injection}\;\mathrm{volume}\;\mathrm{of}\;\mathrm{standard}\;\mathrm{solution}\right)}{V_{2}\left(\mathrm{injection}\;\mathrm{volume}\;\mathrm{of}\;\mathrm{sample}\;\mathrm{solution}\right)}\times V\;(\mathrm{sample}\;\mathrm{volume})}{M(\mathrm{sample}\;\mathrm{amount})}$, X: Contents of 9 compounds in sample solution. ^b^ Not detected.

**Table 8:** α-glucosidase activity inhibition test results.

No.	IC_50_ (μg/mL)
Acarbose	515.98
3	80.35
7	188.42
8	452.50
10	492.11
11	318.44
12	444.88

**Table 9:** Tyrosinase inhibitory activity inhibition test results.

No.	IC_50_ (μg/mL)
Kojic acid	517.07
1	1643.11
8	202.37
11	302.42
12	1750.08

**Table 10:** Acetylcholinesterase inhibitory activity inhibition test results.

No.	IC_50_ (μg/mL)
Huperzine A	502.98
1	476.25
3	504.34
7	491.93
8	500.14
10	521.14
11	516.21
12	507.44

## Supplementary Materials

The following are available online.
