# Supplementary material for: Bioactive Constituents of F. esculentum Bee Pollen and Quantitative Analysis of Samples Collected from Seven Areas by HPLC
Source: Molecules. 2019 Jul 25;24(15):2705. doi: 10.3390/molecules24152705 (PMC6696413; doi:10.3390/molecules24152705)
Supplement: Supplementary file 1 [file molecules-24-02705-s001.pdf]

Supporting Information

Luteolin (1): ESI-MS (negative): *m/z* 285 $[M-H]^-$. ^{1}H-NMR (400 MHz, DMSO-d_6), δ 12.98 (1H, s, 5-OH), 7.43 (1H, br d, 6′-H), 7.41 (1H, br s, 2′-H), 6.90 (1H, d, *J*= 9.0 Hz, 5′-H), 6.46 (1H, d, *J*= 1.8 Hz, 8-H), 6.20 (1H, d, *J*= 1.8 Hz, 6-H), 6.67 (1H, s, 3-H). ^{13}C-NMR (400 MHz, DMSO-d_6): δ 150.15 (C-2), 103.33 (C-3), 182.12 (C-4), 164.35 (C-5), 99.28 (C-6), 164.57 (C-7), 94.29 (C-8), 161.93 (C-9), 104.16 (C-10), 121.96 (C-1′), 116.47 (C-2′), 157.74 (C-3′), 146.19 (C-4′), 113.82 (C-5′), 119.45 (C-6′).

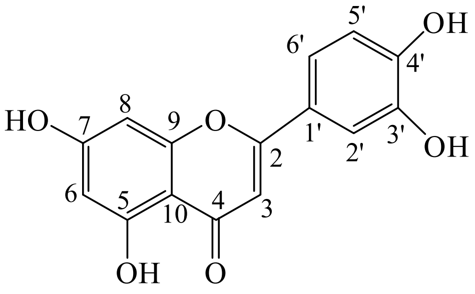

luteolin

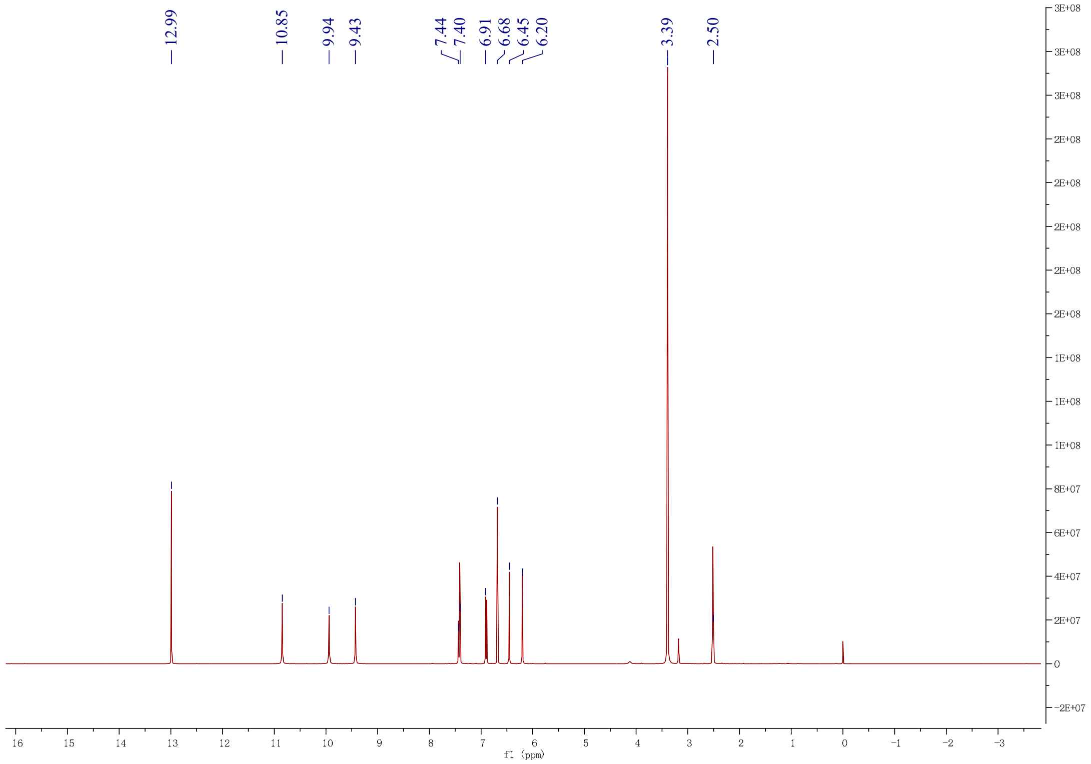

The ^{1}H-NMR spectrum of compound 1

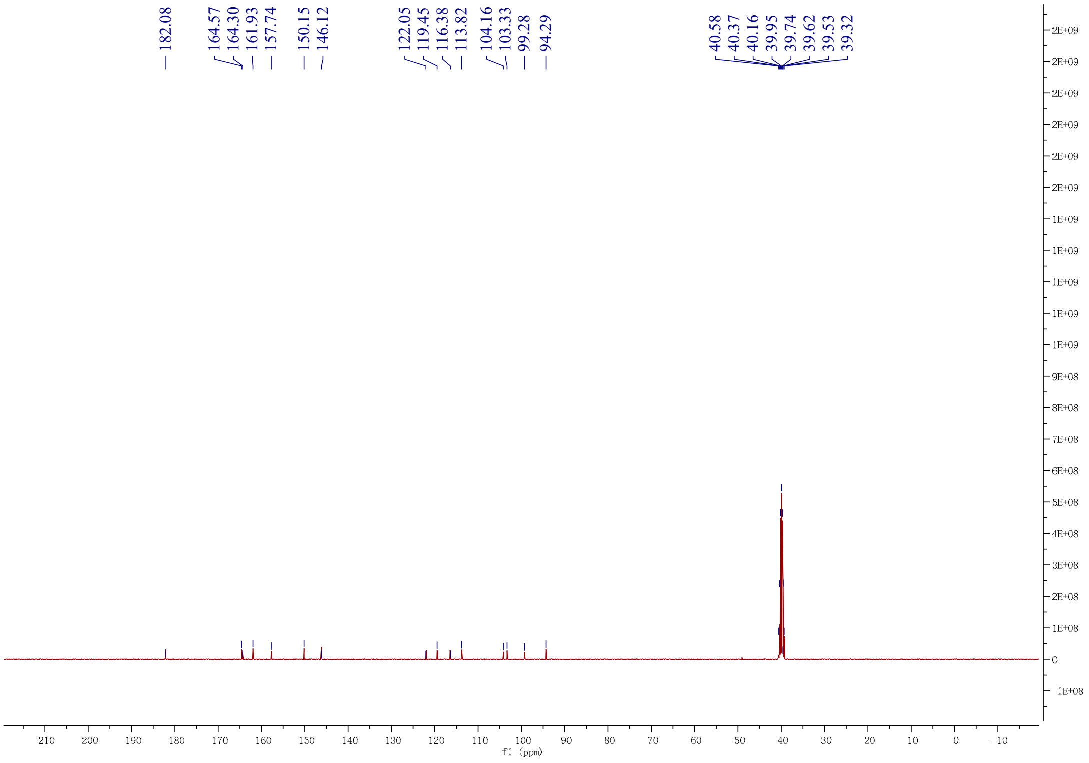

The ^{13}C-NMR spectrum of compound 1

Resveratrol (2): ESI-MS (negative): *m/z* 227 $[M-H]^-$. ^{1}H-NMR (400 MHz, DMSO-d_6), δ 6.38 (1H, d, 1.6 Hz, H-2&6), 6.11 (1H, t, 2.0 Hz, H-4), 7.34 (2H, d, 8.4 Hz, H-2′&6′), 6.75 (2H, d, 8.4 Hz, H-3′&5′), 6.93 (1H, d, 16.4 Hz, H-7′), 6.87 (1H, d, 16.4 Hz, H-8′), 9.24 (s, H-3&5-OH), 9.60 (1H, s, H-4′-OH). ^{13}C-NMR (400 MHz, DMSO-d_6): δ 139.4 (C-1), 104.4 (C-2&6), 158.6 (C-3&5), 101.8 (C-4), 128.1 (C-1′), 127.9 (C-2′&6′), 115.6 (C-3′&5′), 157.3 (C-4′), 127.9 (C-7′), 125.7 (C-8′).

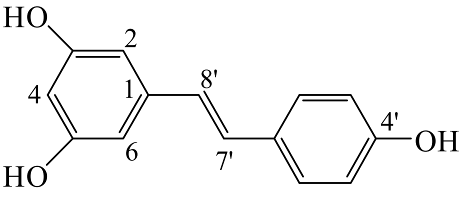

Resveratrol

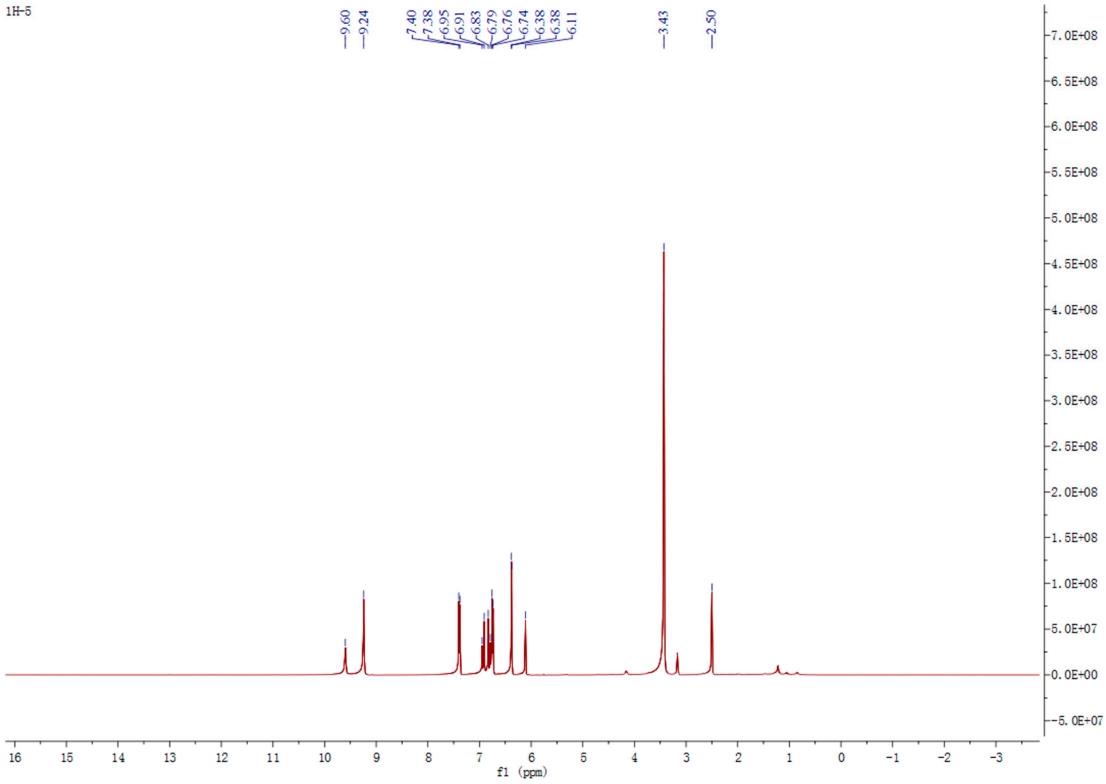

The ^{1}H-NMR spectrum of compound 2

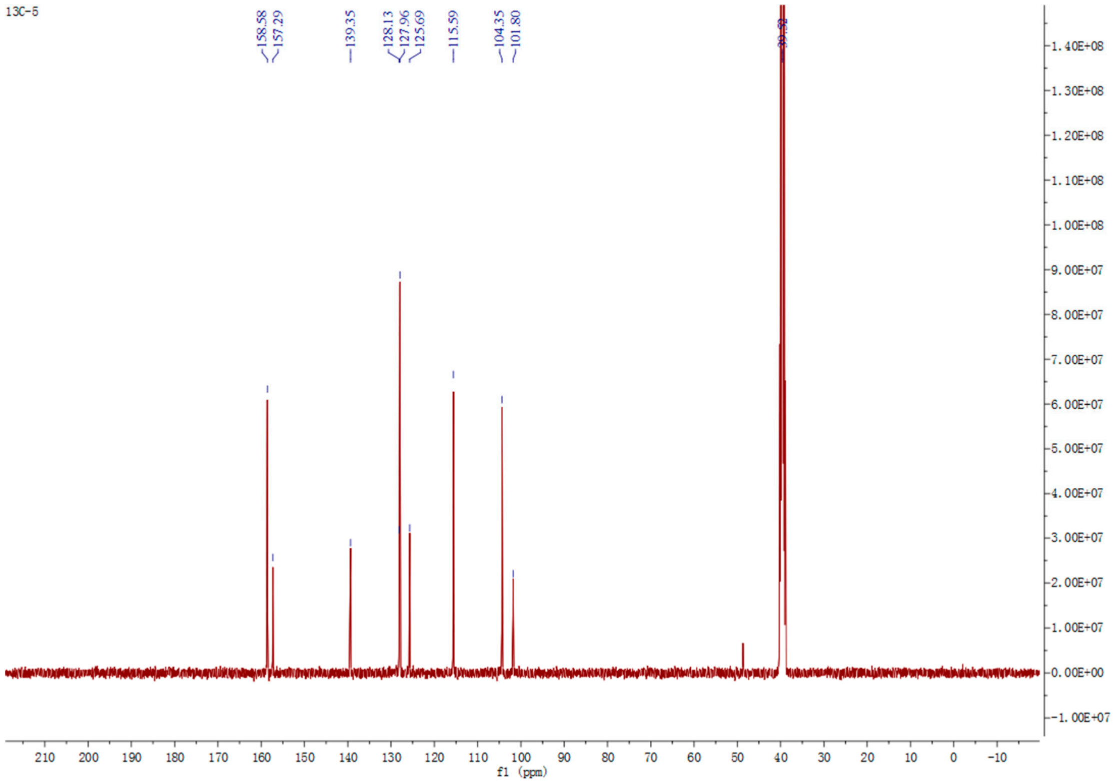

The ^{13}C -NMR spectrum of compound 2

Kaempferol (3): ESI-MS (negative): *m/z* 285 $[M-H]^-$. ^{1}H-NMR (400 MHz, DMSO-d_6): δ 12.49 (1H, s, 5-OH), 10.80 (1H, s, 7-OH), 10.13 (1H, s, 4-OH), 9.43 (1H, s, 3-OH), 8.05 (2H, d, *J*= 8.8Hz, H-2, 6), 6.93 (2H, d, *J*= 8.8 Hz, H-3′, 5′), 6.44 (1H, d, *J*= 1.5 Hz, H-8), 6.19 (1H, d, *J*= 1.5 Hz, H-6). ^{13}C-NMR (400 MHz, DMSO-d_6): δ 147.27 (C-2), 136.12 (C-3), 176.37 (C-4), 161.18 (C-5), 98.66 (C-6), 164.35 (C-7), 93.94 (C-8), 156.64 (C-9), 103.51 (C-10), 122.13 (C-1′), 129.97 (C-2′&6′), 115.90 (C-3′&5′), 159.65 (C-4′).

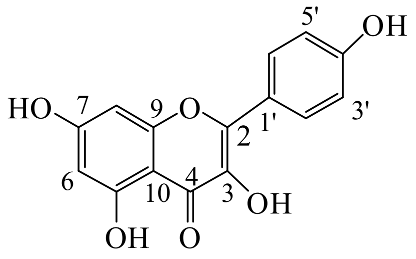

kaempferol

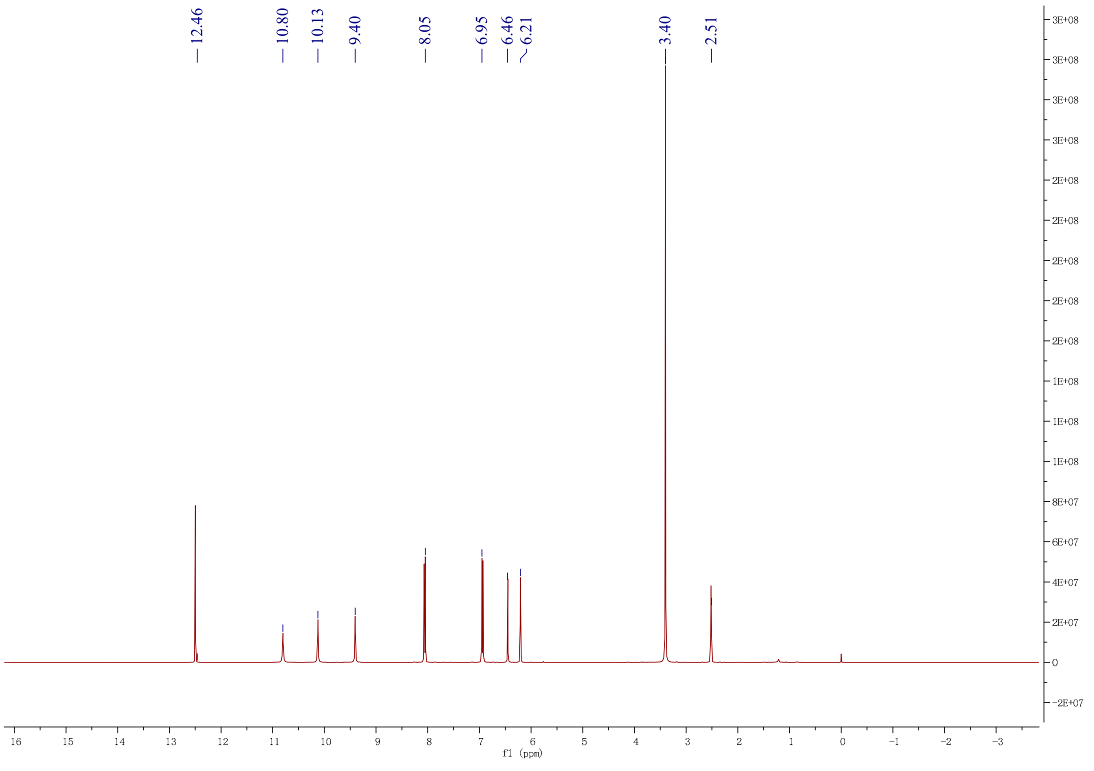

The ^{1}H-NMR spectrum of compound 3

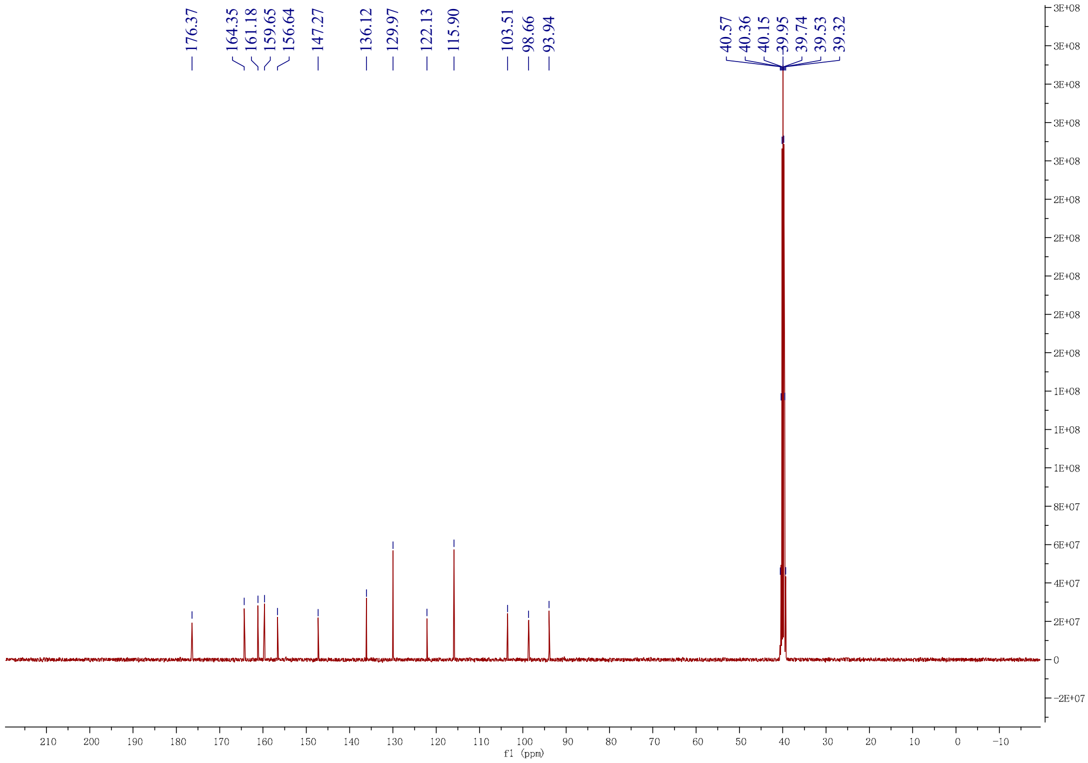

The ^{13}C -NMR spectrum of compound 3

Daucosterol (4): ESI-MS (positive): *m/z* 577 $[M+H]^+$. ^{1}H-NMR (400 MHz, pyridine-d_5): 5.34(1H, m, H-7), 5.06 (2H, d, *J*= 7.71Hz, H-22), 0.91 (3H, s, H-19), 0.89 (3H, s, H-27), 0.87 (3H, d, *J*=7.71 Hz , H-28), 0.85 (3H, m, H-29), 0.83 (3H, m, H-26), 0.63 (3H, s, H-18), 4.58 (1H, d, *J*= 8.25 Hz, H-6). ^{13}C-NMR (400 MHz, DMSO-d_6): δ 37.2 (C-1), 31.6 (C-2), 77.81 (C-3), 39.70 (C-4), 140.65 (C-5), 121.73 (C-6), 31.95 (C-7), 31.80 (C-8), 50.09 (C-9), 36.69 (C-10), 21.05 (C-11), 39.10 (C-12), 42.24 (C-13), 56.58 (C-14), 24.29 (C-15), 19.77 (C-16), 55.98 (C-17), 11.93 (C-18), 19.20 (C-19), 36.17 (C-20), 18.78 (C-21), 33.95 (C-22), 28.33 (C-23), 45.78

(C-24), 29.19 (C-25), 26.09 (C-26), 18.97 (C-27), 23.14 (C-28), 11.75 (C-29), 102.34 (C-1′), 71.43 (C-2′), 75.14 (C-3′), 78.41 (C-4′), 78.33 (C-5′), 62.58 (C-6′).

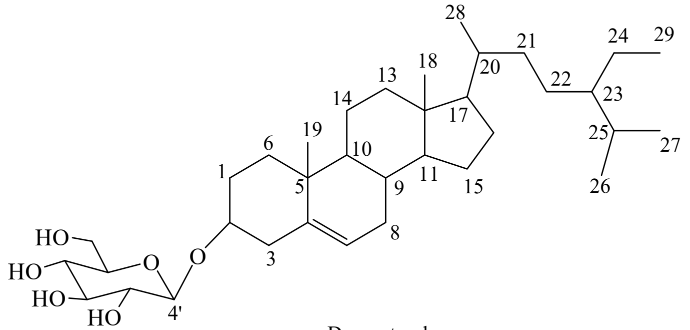

Daucosterol

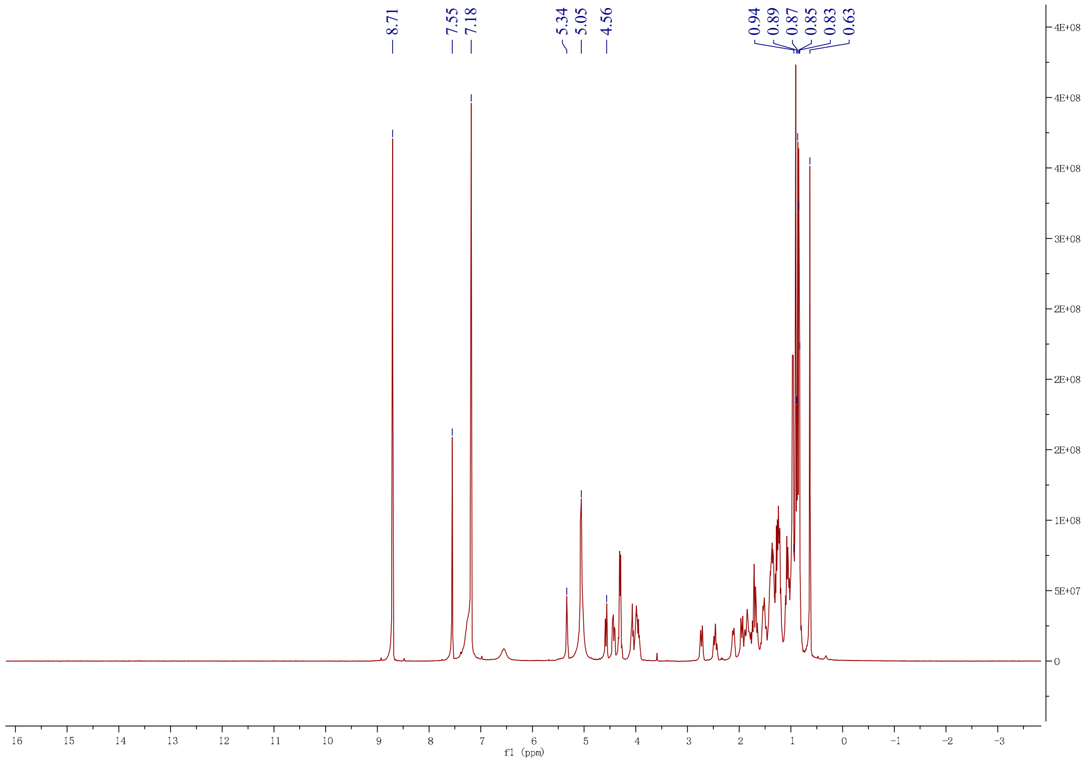

The ^{1}H-NMR spectrum of compound 4

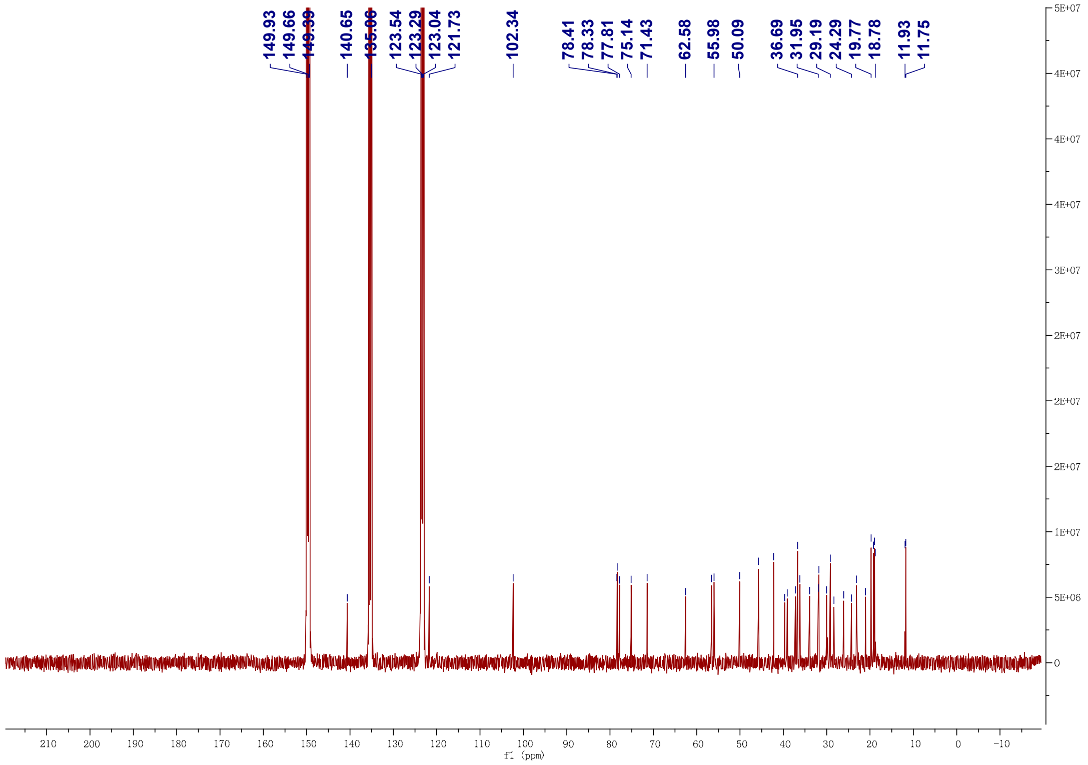

The ^{13}C-NMR spectrum of compound 4

Caffeic acid (5): ESI-MS (negative): *m/z* 179 $[M-H]^-$. ^{1}H-NMR (400 MHz, DMSO-d_6): δ 6.97 (1H, d, *J*= 2.0 Hz, H-3), 7.02 (1H, d, *J*= 2.0 Hz, H-5), 6.95 (1H, d, *J*= 2.0 Hz, H-6), 7.41 (1H, d, *J*= 15.9 Hz, H-7), 6.75 (1H, d, *J*= 8.1 Hz, H-8), 12.16 (1H, s, -COOH), 6.17 (1H, d, *J*= 15.9 Hz, -OH×2). ^{13}C-NMR (400 MHz, DMSO-d_6): δ 127.75 (C-1), 116.46 (C-2), 146.77 (C-3), 149.44 (C-4), 115.04 (C-5), 122.86 (C-6), 147.05 (C-7), 115.47 (C-8), 171.04 (C-9).

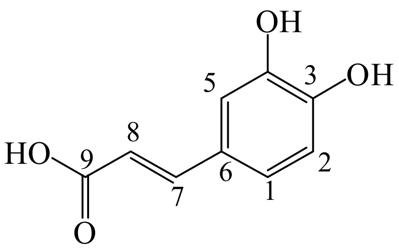

caffeic acid

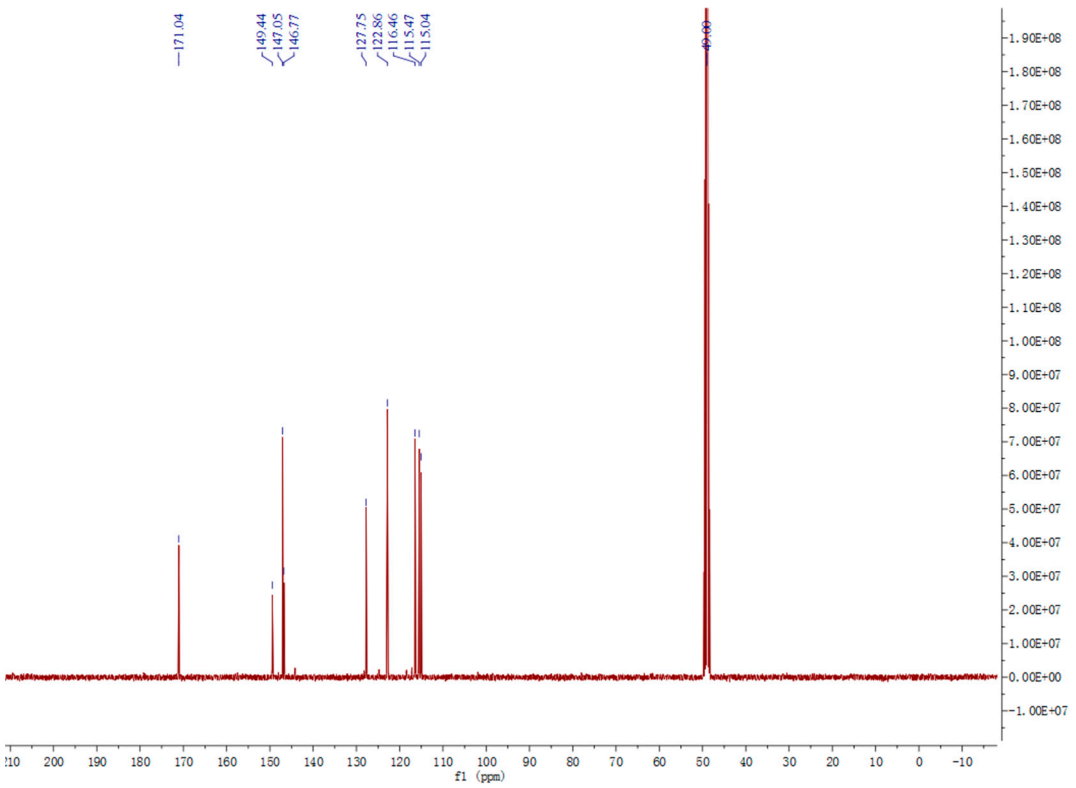

The ^{13}C-NMR spectrum of compound 5

Chlorogenic acid (6): ESI-MS (negative): *m/z* 353 [M-H]$^-$. ^{1}H-NMR (400 MHz, methanol-d_4): δ 7.48 (1H, d, *J*= 15.9 Hz, H-7), 6.97 (1H, d, *J*= 2.0 Hz, H-6), 6.88 (1H, dd, *J*= 2.0, 8.1 Hz, H-2), 6.70 (1H, d, *J*= 8.2 Hz, H-5), 6.19 (1H, d, *J*= 15.9 Hz, H-8), 5.25 (1H, d, *J*= 4.2 Hz, H-5′), 4.08 (1H, s, H-1′), 3.65 (dd, *J*= 8.4, 3.1 Hz, H-6′), 3.30~3.20 (2H, m, H-2′), 2.03 (2H, d, *J*= 5.2 Hz, H-4′). ^{13}C-NMR (400 MHz, DMSO-d_6): δ 127.74 (C-1), 115.12 (C-2), 149.58 (C-3), 147.08 (C-4), 116.43 (C-5), 123.00 (C-6), 146.79 (C-7), 115.19 (C-8), 168.62 (C-9), 71.95 (C-1′), 38.17 (C-2′), 73.38 (C-3′), 38.69 (C-4′), 71.22 (C-5′), 76.09 (C-6′), 177.03 (C-7′).

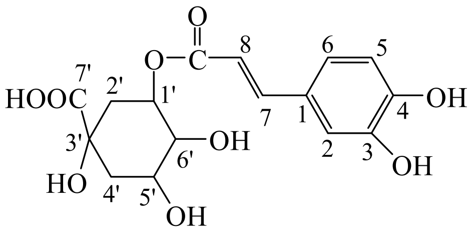

chlorogenic acid

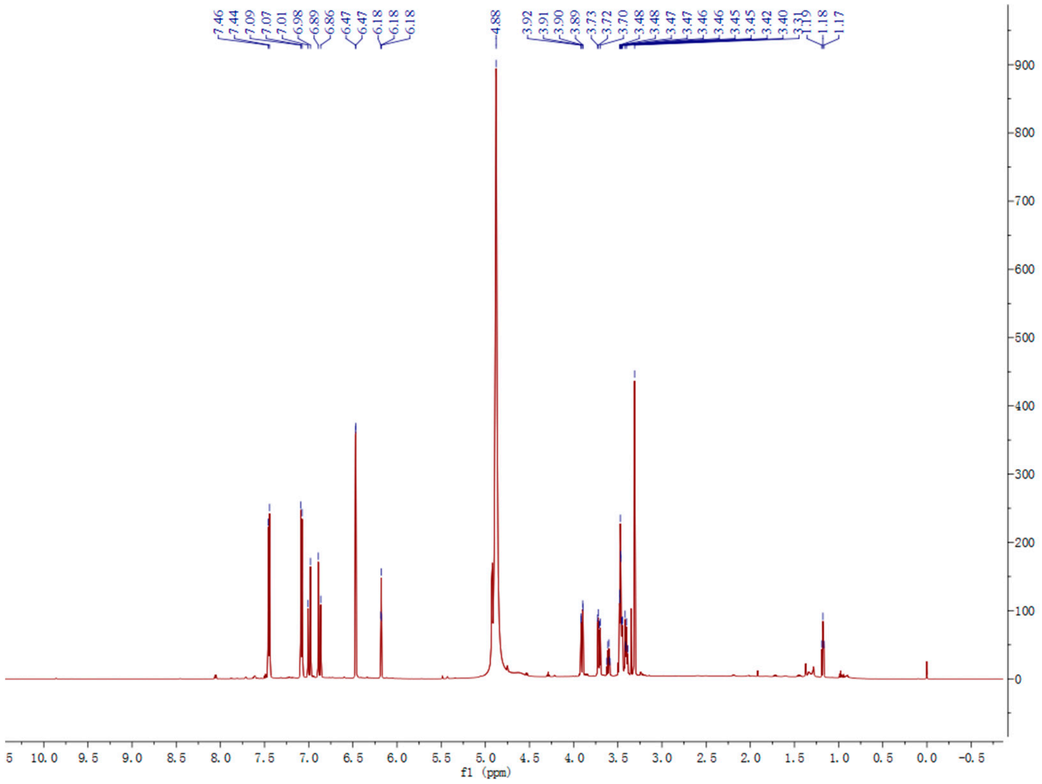

The ^{1}H-NMR spectrum of compound 6

Rutin (7): ESI-MS (negative): *m/z* 609 $[M-H]^-$. ^{1}H-NMR (400 MHz, methanol-d_4): δ 12.61 (1H, s, 5-OH), 6.20 (1H, d, *J*= 1.9 Hz, 6-OH), 6.39 (1H, d, *J*= 1.9 Hz, 8-H), 7.54 (1H, s, 2′-H), 6.84 (1H, d, *J*= 9.0 Hz, 5′-H), 7.56 (1H, d, *J*= 9.0Hz, 6′-H), δ 5.34 (1H, d, *J*= 7.0 Hz, 1″-H), 4.39 (1H, br s, 1‴-H), 1.00 (3H, d, *J*= 6.0 Hz, -CH_3). ^{13}C-NMR (400 MHz, DMSO-d_6): δ 157.09 (C-2), 134.23 (C-3), 178.00 (C-4), 161.57 (C-5), 98.52 (C-6), 164.00 (C-7), 93.45 (C-8), 157.72 (C-9), 104.21 (C-10), 121.70 (C-1′), 122.15 (C-2′), 116.28 (C-3′), 148.40 (C-4′), 144.43 (C-5′), 114.63 (C-6′), 101.01 (C-1″), 74.32 (C-2″), 76.76 (C-3″), 69.97 (C-4″), 75.79 (C-5″), 67.14 (C-6″), 103.22 (C-1‴), 70.67 (C-2‴), 70.81 (C-3‴), 72.52 (C-4‴), 68.30 (C-5‴), 15.92 (C-6‴).

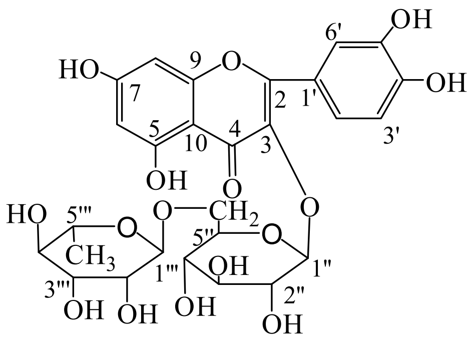

rutin

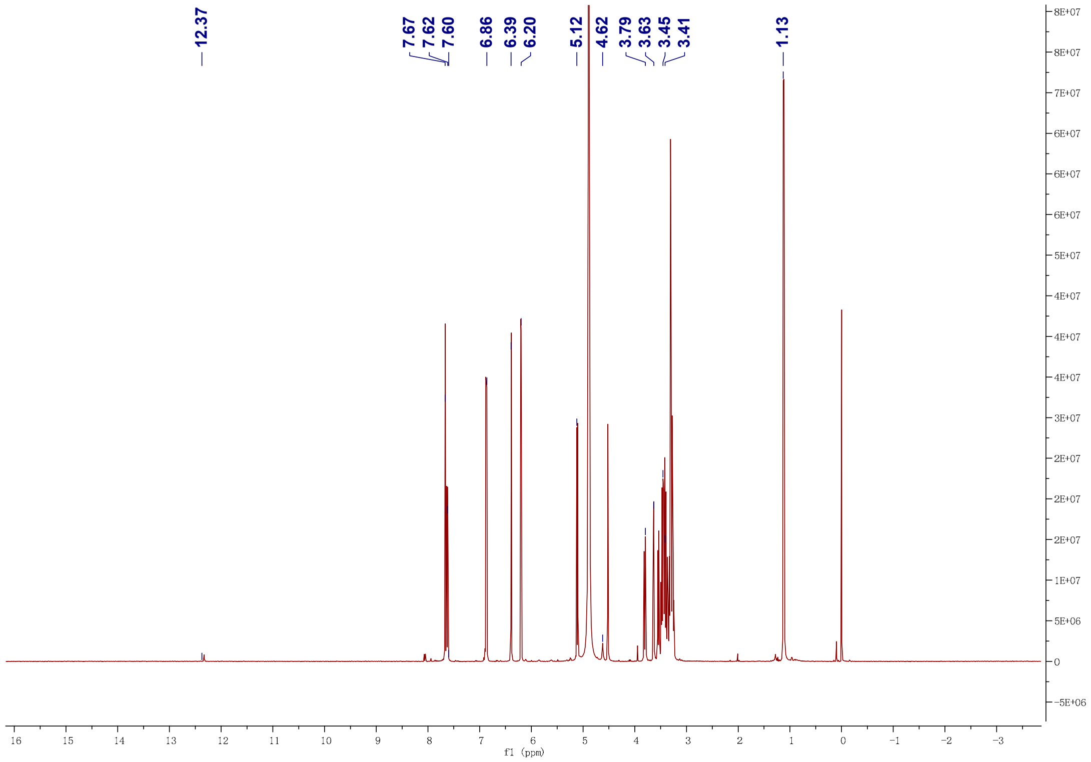

The ^{1}H-NMR spectrum of compound 7

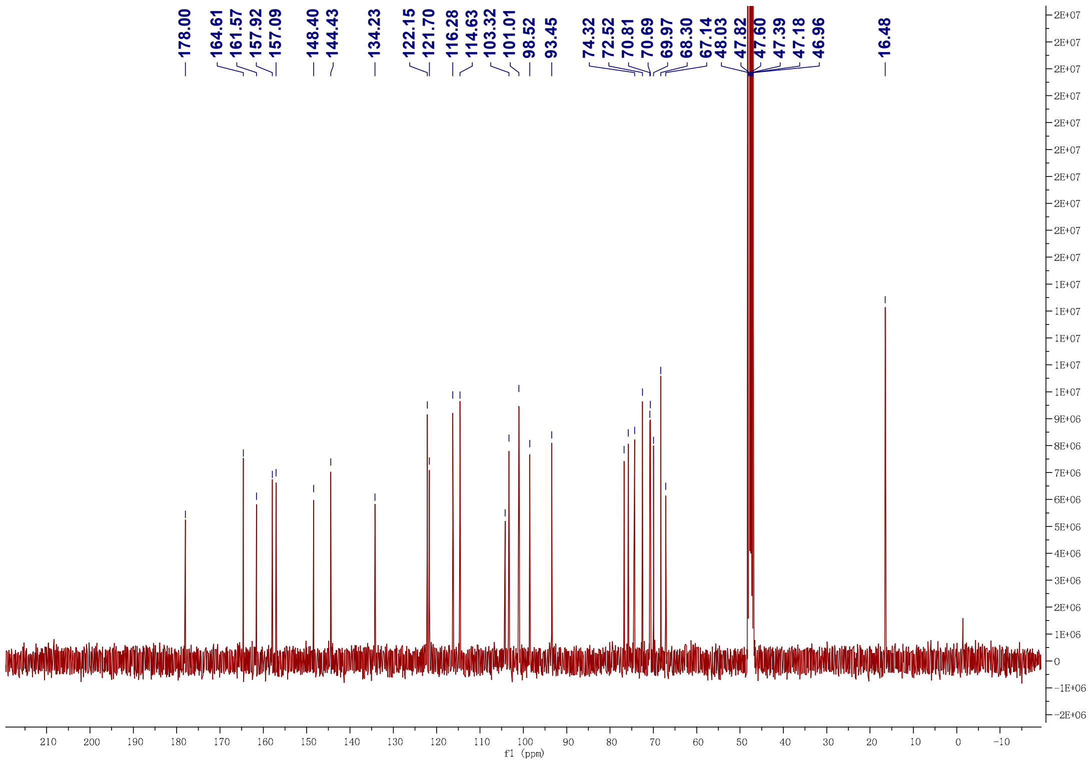

The ^{13}C-NMR spectrum of compound 7

Ergosterol peroxide (8): ESI-MS (positive): *m/z* 429 $[M+H]^+$. ^{1}H-NMR (400 MHz, methanol-d_4): δ 4.39 (m, H-3), 6.34 (d, *J*= 8.4 Hz, H-6), 6.54 (d, *J*= 8.4Hz, H-7), 0.77 (s, H-18), 0.88 (s, H-19), 1.01 (d, *J*= 6.6 Hz, H-21), 5.20(dd, *J*= 15.3, 8.0 Hz, H-22&23), 0.86 (d, *J*= 6.6Hz, H-26), 0.85 (d, *J*= 6.6 Hz, H-27), 1.02 (d, *J*= 6.8 Hz, H-28). ^{13}C-NMR (400 MHz, DMSO-d_6): δ 35.15 (C-1), 31.94 (C-2), 64.87 (C-3), 28.82 (C-4), 82.47 (C-5), 136.49 (C-6), 129.48 (C-7), 80.81 (C-8), 54.00 (C-9), 36.52 (C-10), 25.52 (C-11), 38.76 (C-12), 45.99 (C-13), 53.33 (C-14), 20.18 (C-15), 29.03 (C-16), 56.70 (C-17), 12.84 (C-18), 19.05 (C-19), 40.32 (C-20), 22.32 (C-21), 136.20 (C-22), 133.08 (C-23), 44.89 (C-24), 32.80 (C-25), 19.84 (C-26), 19.06 (C-27), 16.99 (C-28).

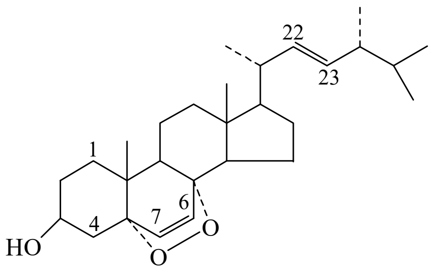

Ergosterol peroxide

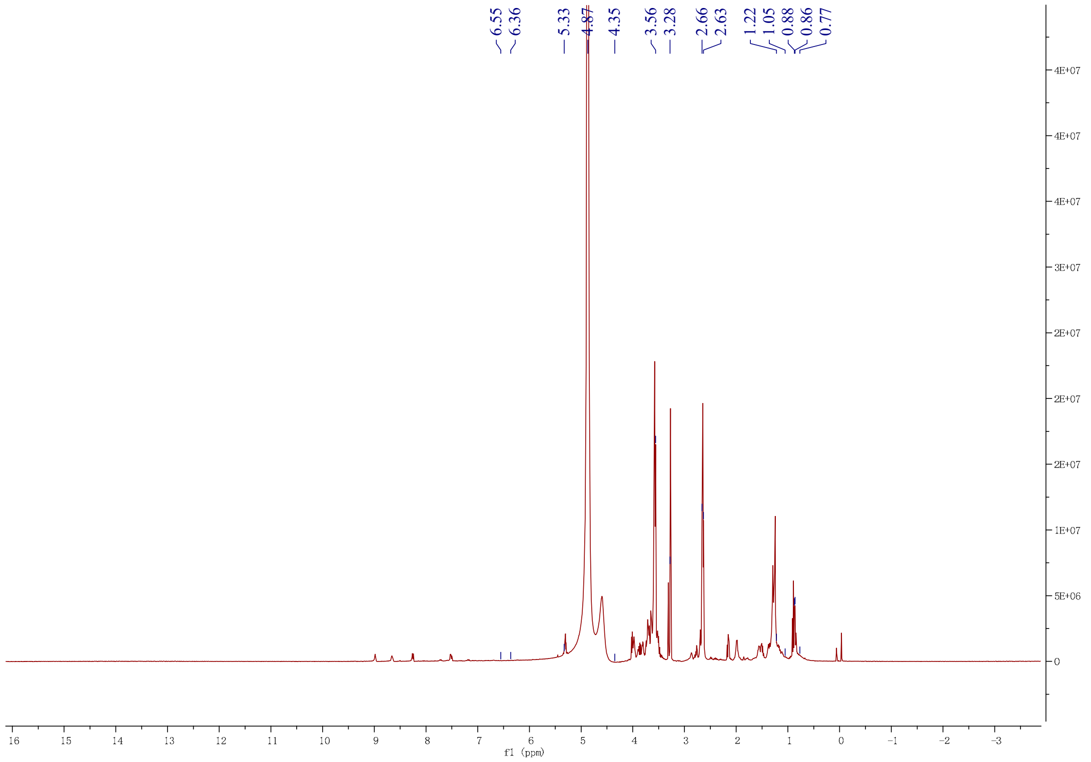

The ^{1}H-NMR spectrum of compound 8

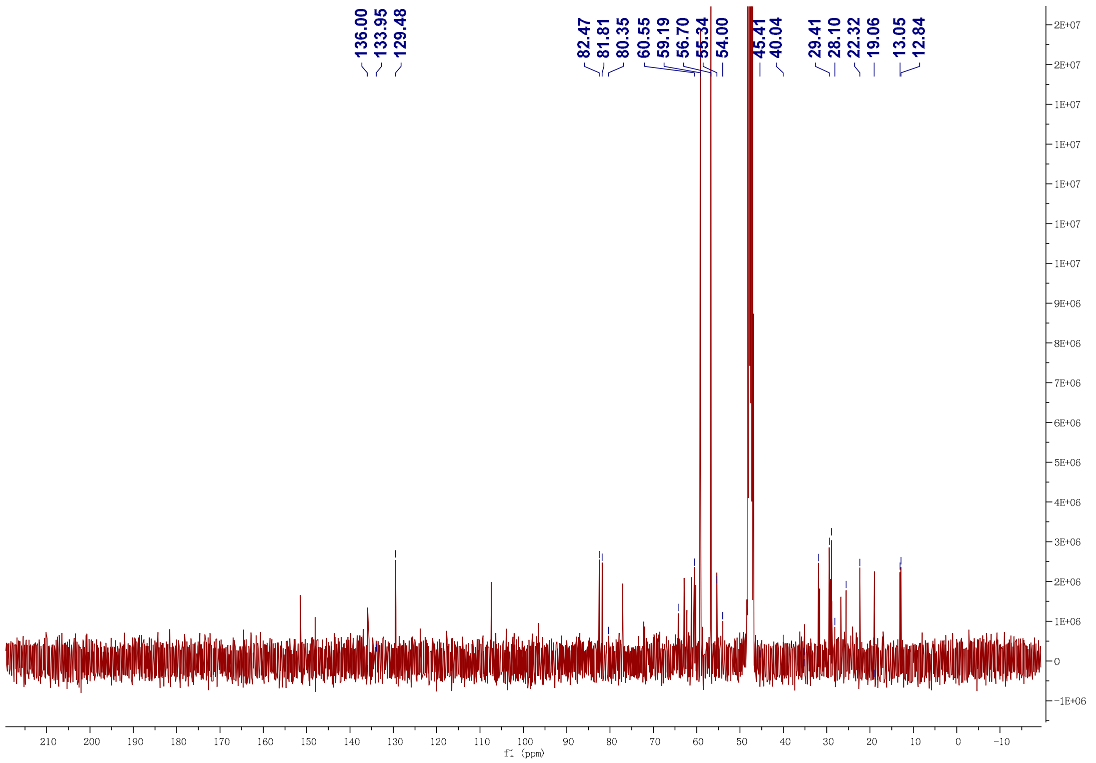

The ^{13}C-NMR spectrum of compound 8

Octacosanol (9): ESI-MS (positive): *m/z* 411 [M+H]$^+$. ^{1}H-NMR (400 MHz, DMSO-d$_6$): δ 3.64 (2H, m, *J*= 6.8 Hz, H-1), 1.55 (2H, m, H-2), 1.28 (50H, brs. H-3-27), 0.88 (3H, t, *J*= 6.8 Hz, H-28). ^{13}C-NMR (400 MHz, DMSO-d6): δ 64.1 (C-1), 30.9 (C-2), 28.6, 28.15, 27.94 (C-4−26), 24.7 (C-3), 21.7 (C-27), 13.1 (C-28).

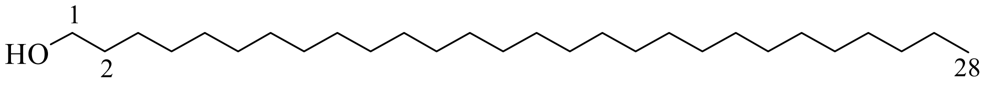

Octacosanol

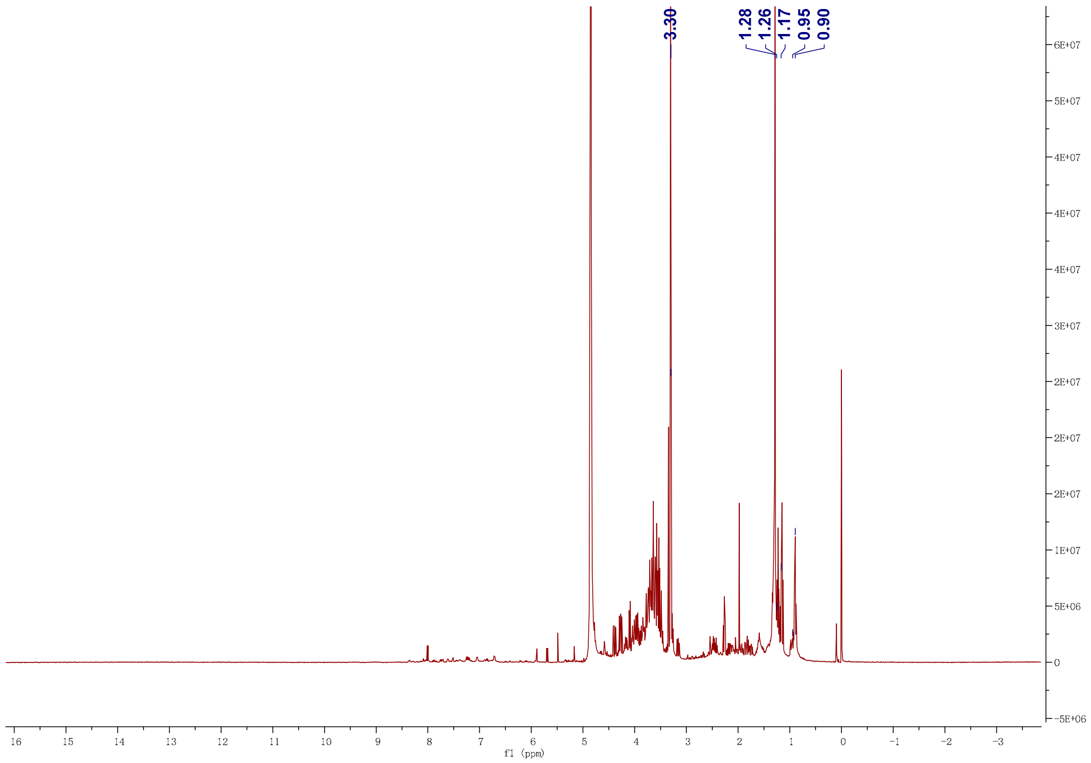

The ^{1}H-NMR spectrum of compound 9

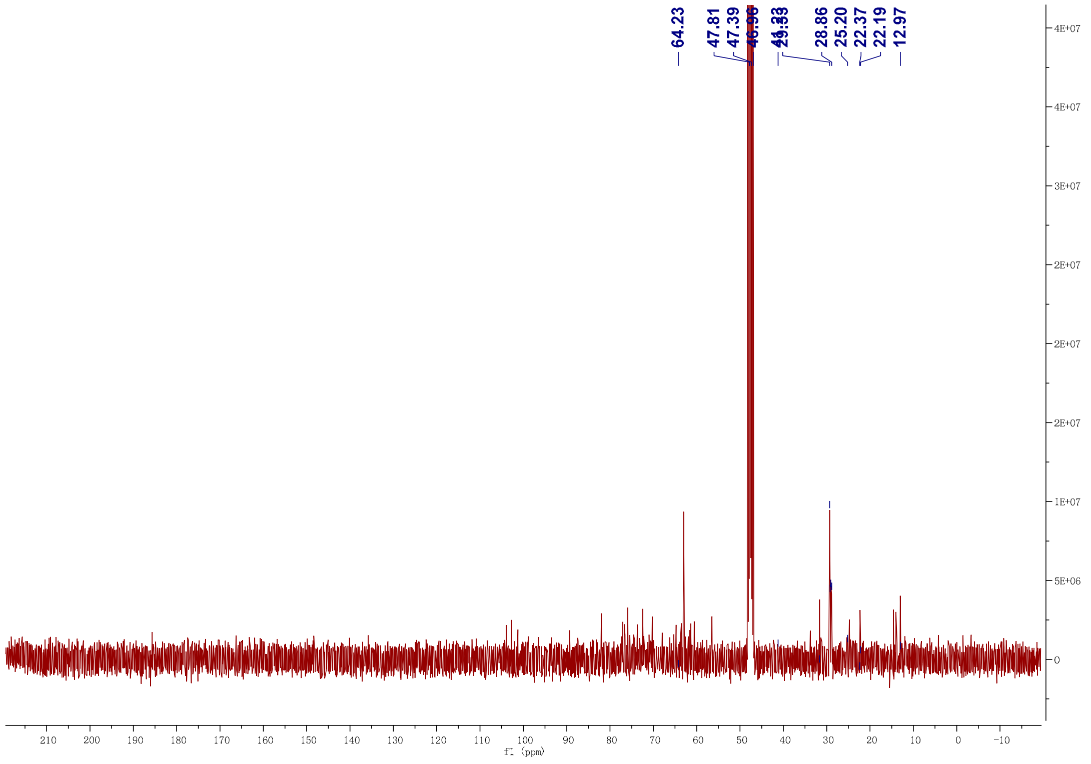

The ^{13}C-NMR spectrum of compound 9

2-β-D-glucopyranosyloxy-1-hydroxy-trideca-3,5,7,9,11-pentayne (10): ESI-MS (positive): *m/z* 359 $[M+H]^+$. ^{1}H-NMR (400 MHz, DMSO-d_6): δ 3.58 (1H, m, H-1a), 3.40 (1H, m, H-1b), 4.8 (1H, t, *J*= 5.7 Hz, H-2), 2.09 (3H, s, H-13), 4.59 (1H, d, *J*= 6.5 Hz, H-1′), 3.07(1H, t, *J*= 8.9 Hz, H-2′), 3.03 (1H, t, *J*= 8.9 Hz, H-3′), 3.00 (1H, t, *J*= 8.9 Hz, H-4′), 3.09 (1H, m, H-5′), 3.48 (2H, m, H-6'a/b, H-6′). ^{13}C-NMR (400 MHz, DMSO-d_6): δ 62.02 (C-1), 69.01 (C-2), 69.18 (C-3), 60.67 (C-4), 60.94 (C-5), 62.22 (C-6), 62.42 (C-7), 63.22 (C-8), 63.61 (C-9), 76.52 (C-10), 59.69 (C-11), 80.1 (C-12), 4.2 (C-13), 100.00 (C-1′), 72.65 (C-2′), 76.08 (C-3′), 69.48 (C-4′), 75.21 (C-5′), 63.13 (C-6′).

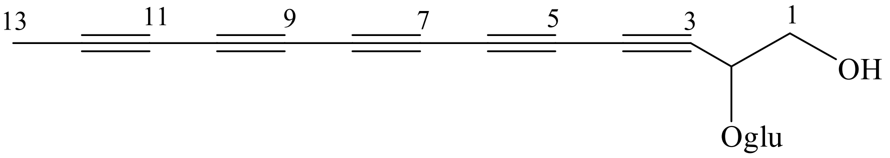

2-β-D-glucopyranosyloxy-1-hydroxy-trideca-3,5,7,9,11-pentayne

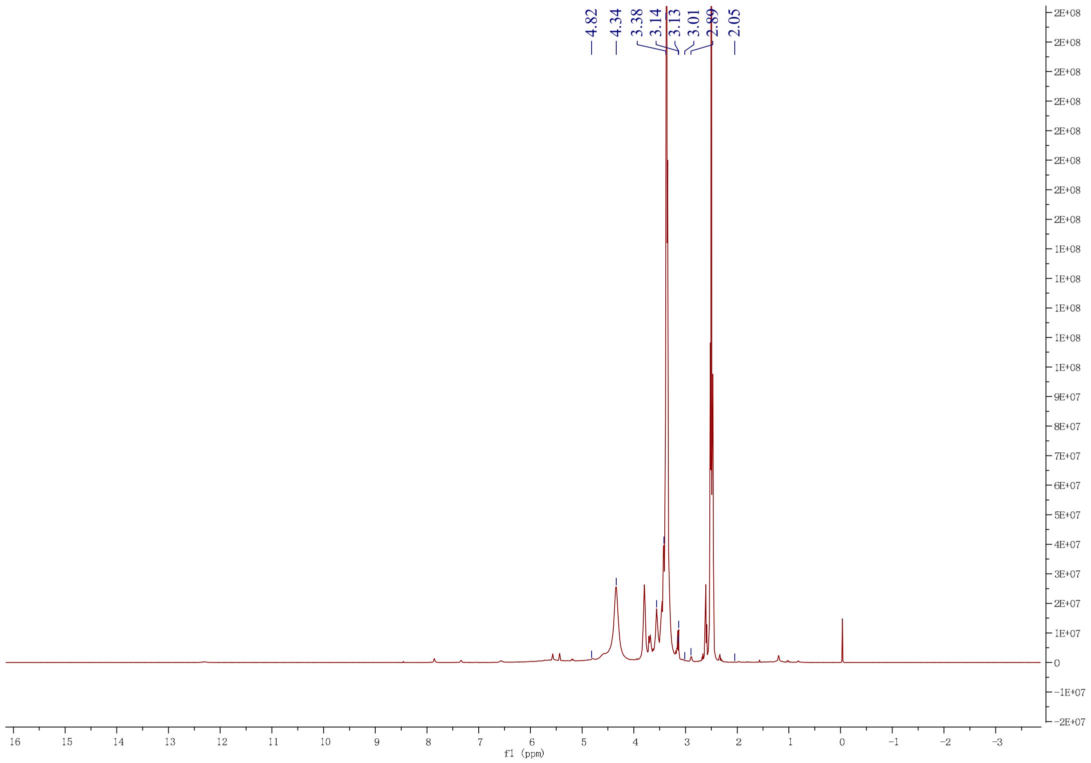

The ^{1}H-NMR spectrum of compound 10

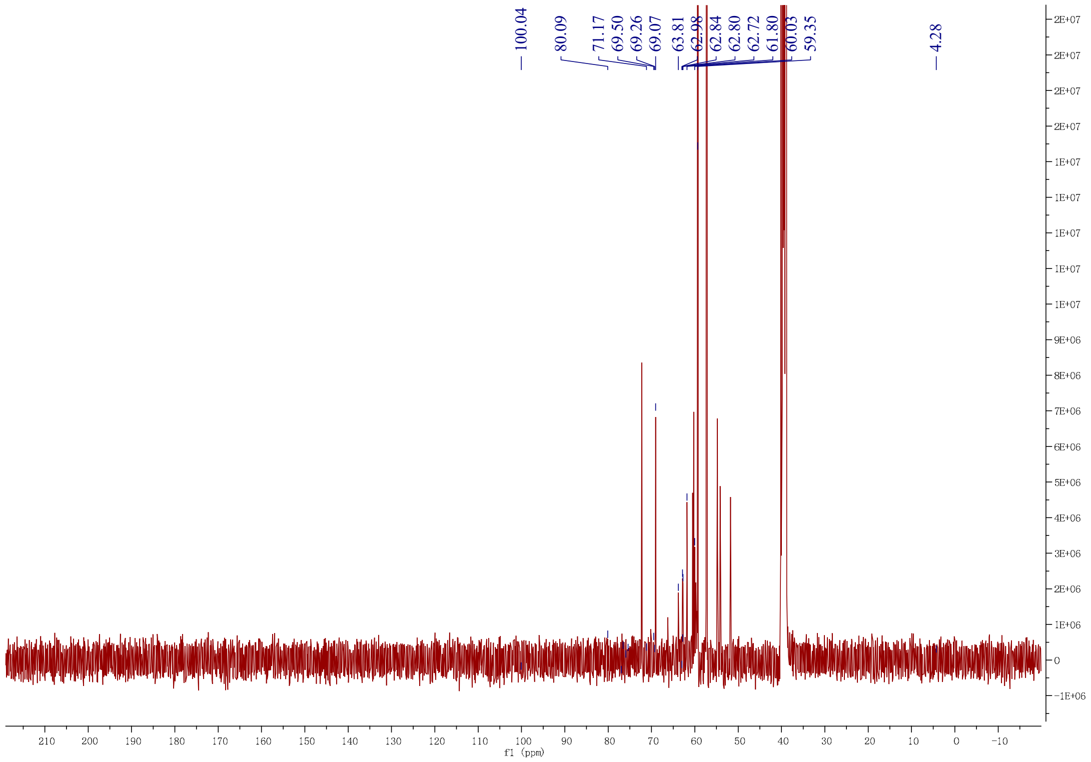

The ^{13}C-NMR spectrum of compound 10

α-D-Altp-OMe(N2-6)α-D-Glep-Ome (11): ESI-MS (positive): *m/z* 370 $[M+H]^+$. ^{1}H-NMR (400 MHz, methanol-d$_4$): δ 4.82 (H-1), 3.57 (H-2), 3.65 (H-3), 3.29 (H-4), 3.71 (H-5), 2.80 (H-6R), 3.09 (H-6S), 3.50 (H-OMe), 4.71 (H-1′), 3.02 (H-2′), 4.00 (H-3′), 3.85 (H-4′), 3.96 (H-5′), 3.79 (H-6R), 3.89 (H-6S), 3.44 (H-OMe). ^{13}C-NMR (400 MHz, Methanol-*d$_4$*): δ 100.00 (C-1), 72.05 (C-2), 72.00 (C-3), 72.25 (C-4), 70.88 (C-5), 48.99 (C-6), 56.81 (C-OMe), 102.30 (C-1′), 60.57 (C-2′), 68.94 (C-3′), 64.29 (C-4′), 69.53 (C-5′), 62.26 (C-6′), 55.34 (C-OMe).

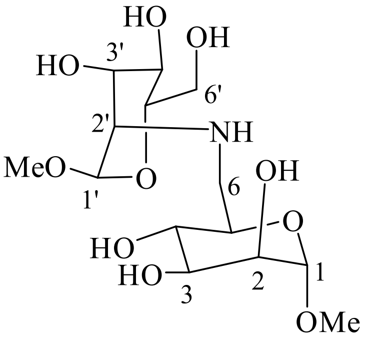

α-D-Altp-OMe（N2-6）α-D-Glep-OMe

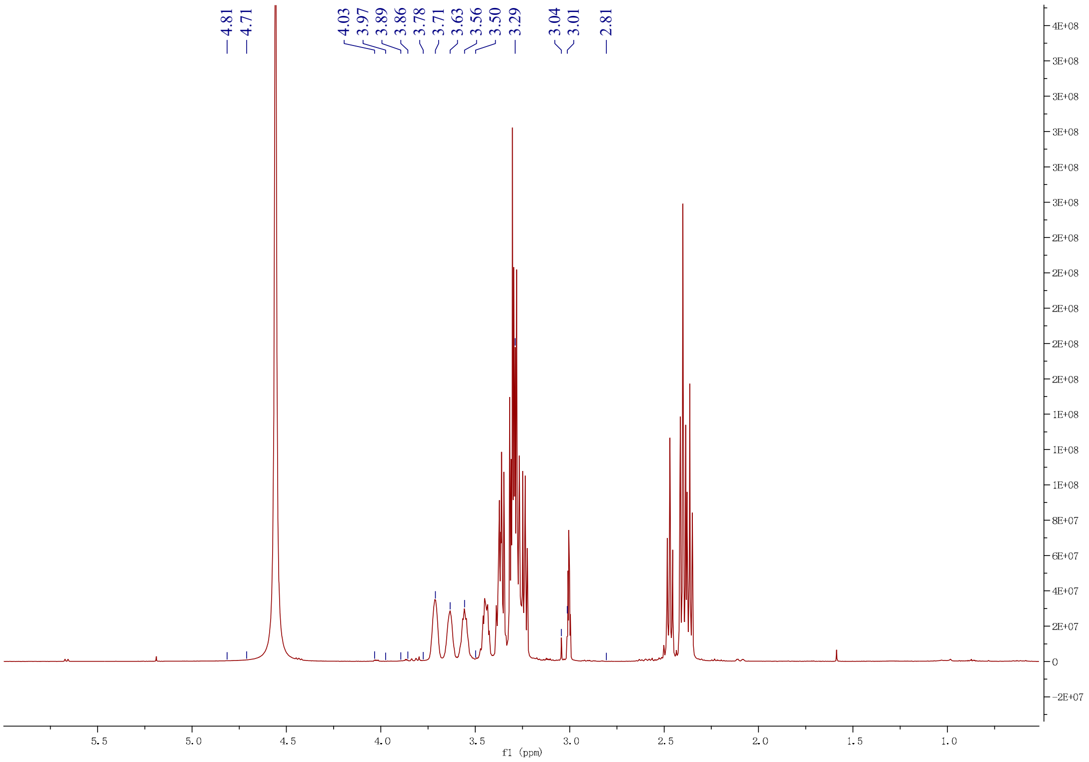

The ^{1}H-NMR spectrum of compound 11

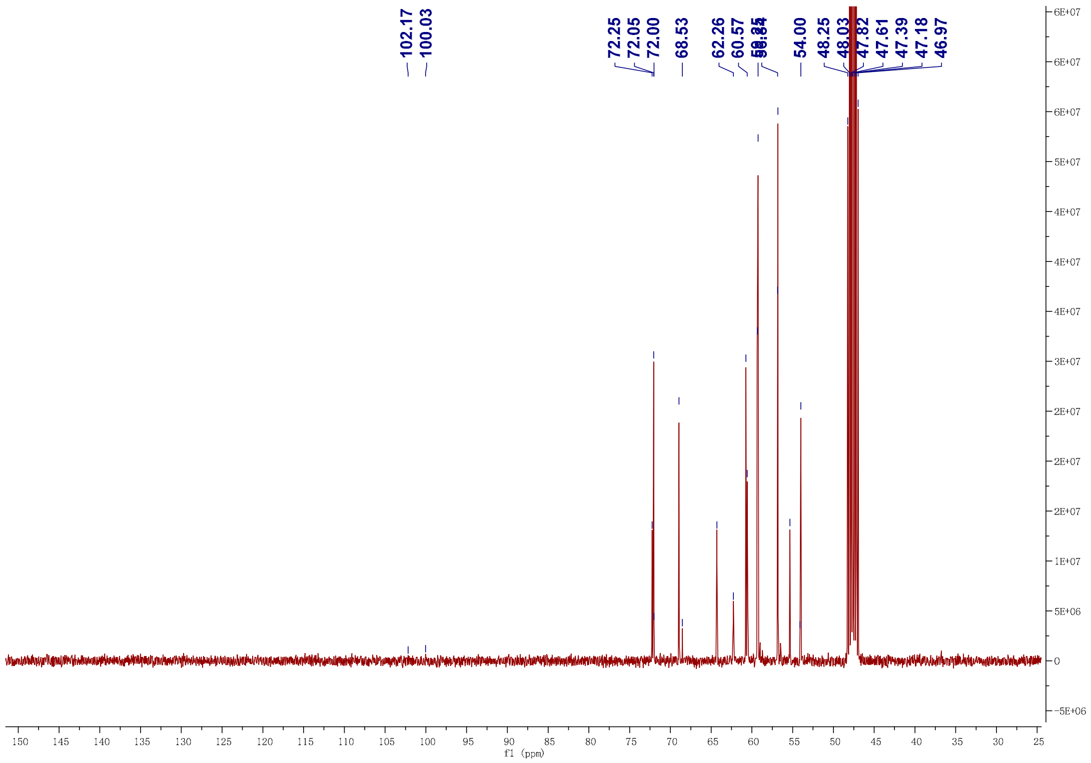

The ^{13}C-NMR spectrum of compound 11

O-α-D-glucopyranosyl-(1→4)-α-D-glucopyranosyl-(1→4)-α-D-glucopyranosyl-(1→2)-β-D-fructofuranoside (12): ESI-MS (positive): *m/z* 667 $[M+H]^+$. ^{13}C-NMR (400 MHz, methanol-d_4): δ 103.90 (C-1), 70.25 (C-2), 77.03 (C-3), 68.01 (C-4), 75.89 (C-5), 60.56 (C-6), 98.46, 101.33 (C-1′), 70.46 (C-2′), 73.75, 73.68 (C-3′), 71.00, 72.11 (C-4′), 72.20, 72.46 (C-5′), 61.37, 61.32 (C-6′), 63.01 (C-1″), 102.70 (C-2″), 76.70 (C-3″), 76.50 (C-4″), 81.98 (C-5″), 63.53 (C-6″).

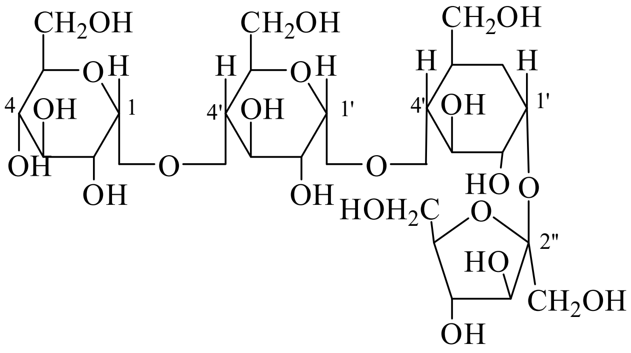

O-α- D -glucopyranosyl-(1→4)-α-D-glucopyranosyl-(1→4)-α-D-glucopyranosyl-(1→2)-D-fructofuranoside

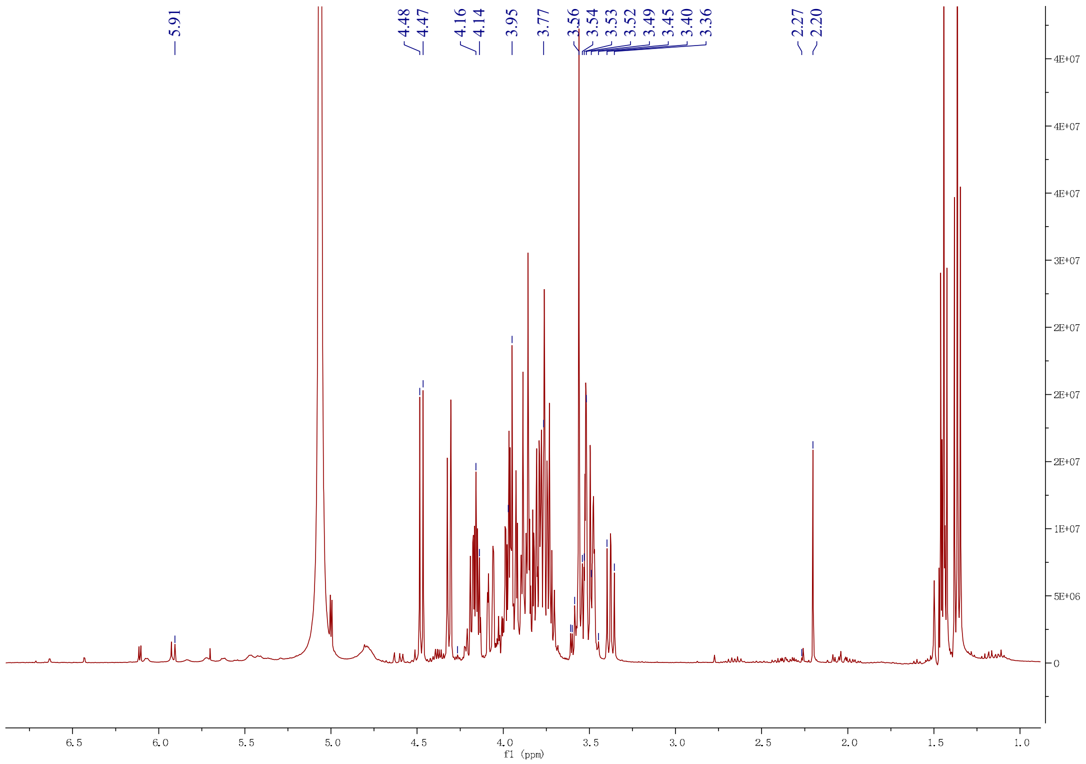

The ¹H-NMR spectrum of compound 12

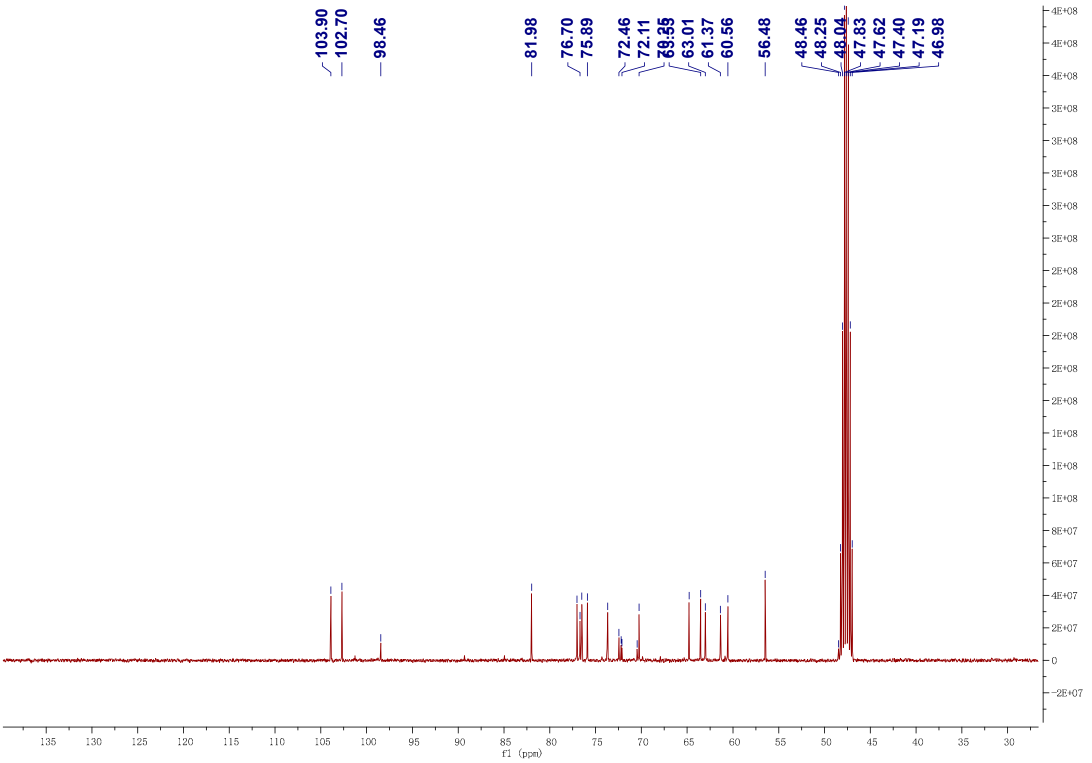

The ¹³C-NMR spectrum of compound 12

(-)-catechin (13): ESI-MS (positive): *m/z* 291 $[M+H]^+$. ^{13}C-NMR (400 MHz, DMSO-d_6): δ 83.20 (C-2), 68.97 (C-3), 28.99 (C-4), 188.99 (C-5), 96.17 (C-6), 184.87 (C-7), 95.46 (C-8), 108.21 (C-9), 100.80 (C-10), 132.51 (C-1′), 116.38 (C-2′), 157.76 (C-3′), 146.42 (C-4′), 115.19 (C-5′), 120.26 (C-6′).

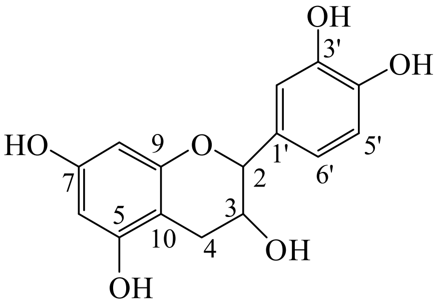

catechin

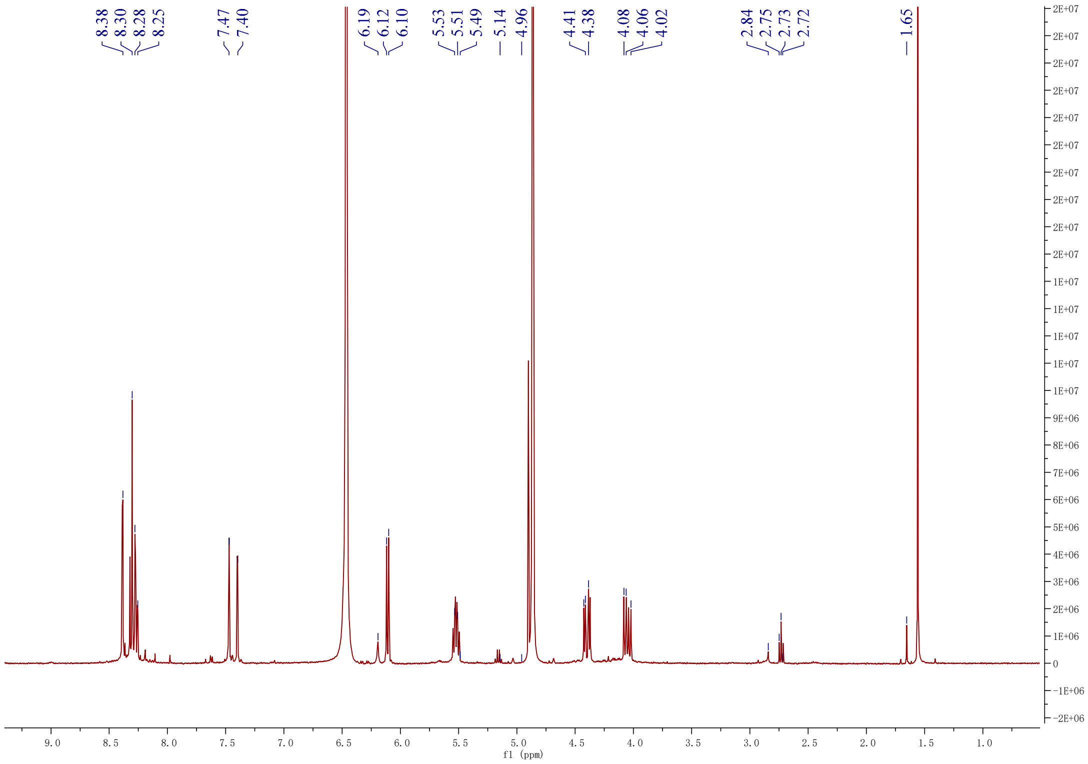

The ^{1}H-NMR spectrum of compound 13

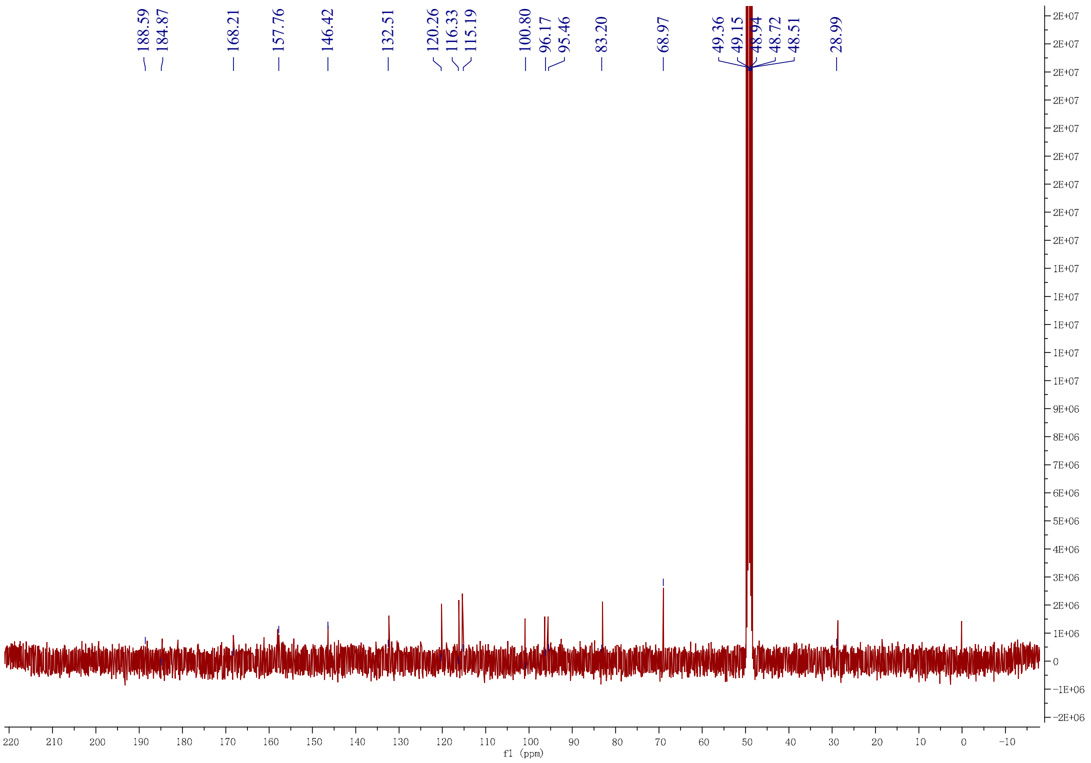

The ^{13}C-NMR spectrum of compound 13

Quercetin (14): ESI-MS (positive): *m/z* 449 $[M+H]^+$. ^{1}H-NMR (400 MHz, DMSO-d_6): δ 7.36 (1H, dd, *J*= 2 Hz, 8 Hz, H-6′), 7.20 (1H, d, *J*= 2.0 Hz, H-2′), 6.96 (1H, d, *J*= 8.0 Hz, H-5′), 6.22 (1H, d, *J*= 2.0 Hz, H-8), 6.41 (1H, d, *J*= 2.0 Hz, H-6), 5.20 (1H, d, *J*= 2.0 Hz, H-1″), 0.98 (3H, d, *J*= 9.0Hz, CH_3), 3.07-3.95 (4H, m, H-2″,3″,4″,5″). ^{13}C-NMR (400 MHz, DMSO-d_6): δ 157.5 (C-2), 136.5 (C-3), 179.0 (C-4), 163.9 (C-5), 98.7 (C-6), 164.7 (C-7), 95.1 (C-8), 159.8 (C-9), 104.9 (C-10), 123.5 (C-1′), 117.4 (C-2′), 145.9 (C-3′), 150.2 (C-4′), 116.9 (C-5′), 123.1 (C-6′), 101.9 (C-1″), 71.9 (C-2″), 72.3 (C-3″), 73.5 (C-4″), 71.0 (C-5″), 18.01 (C-6″).

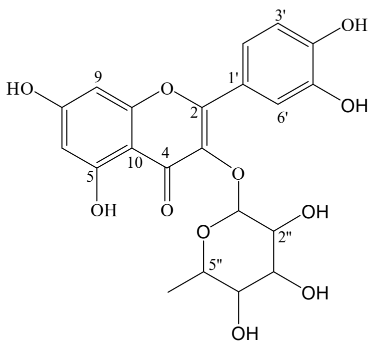

Quercetin

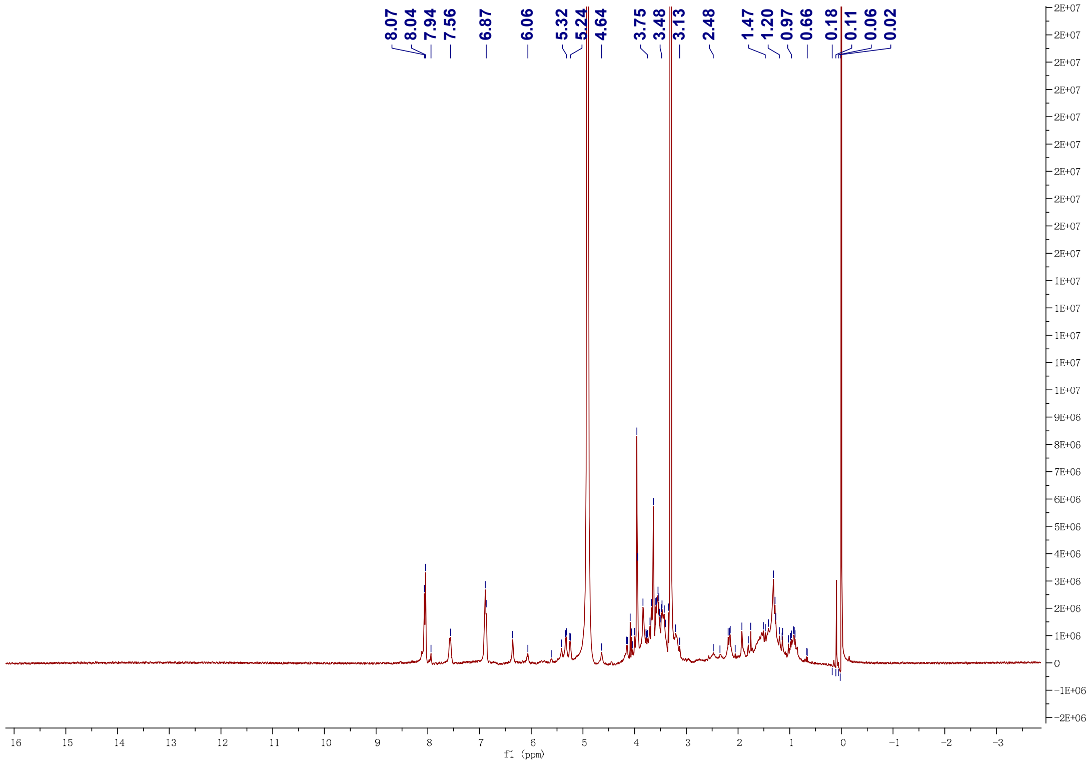

The ^{1}H-NMR spectrum of compound 14

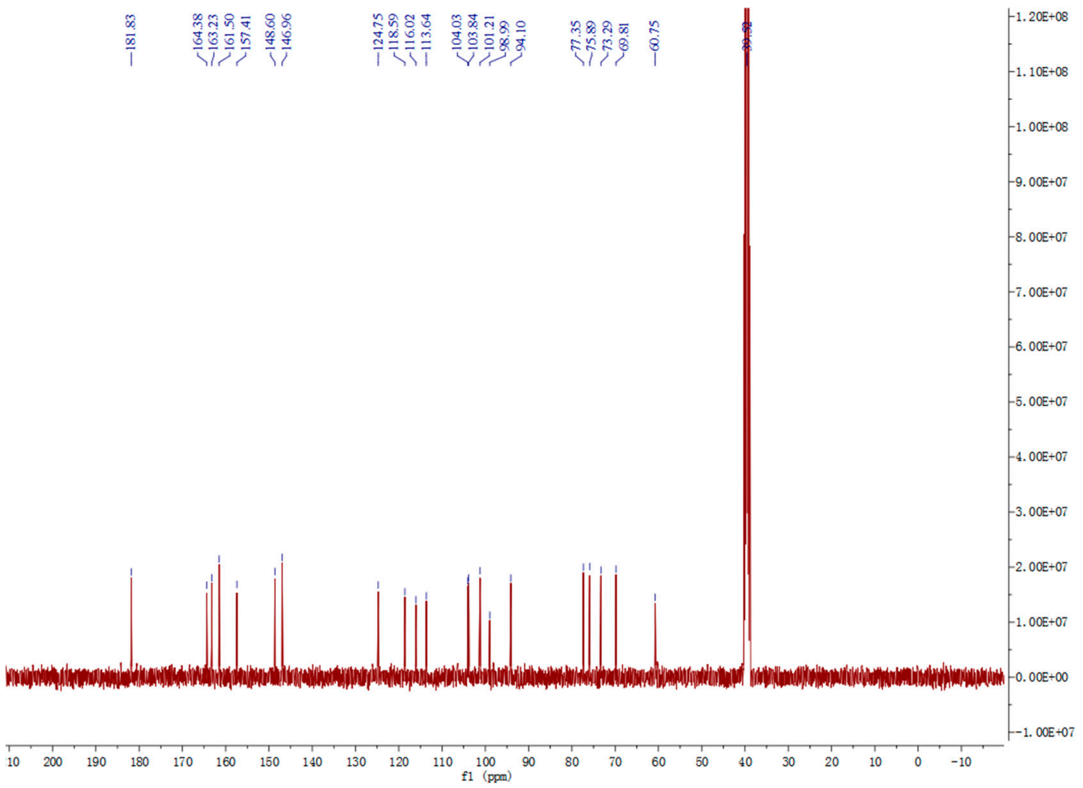

The ^{13}C-NMR spectrum of compound 14

Oleanolic acid (15): ESI-MS (positive): *m/z* 455 [M-H]$^-$. ^{1}H-NMR (400 MHz, DMSO-d_6): δ 1.57 (1H, m, 1-H$_A$), 1.02 (1H, s, 1-H$_B$), 1.82 (1H, m, 2-H), 3.44 (1H, dd, *J*= 5.4, 10.6 Hz, 3-H), 0.88 (1H, d, *J*= 10.5 Hz, 5-H), 1.58 (1H, m, 6-H$_A$), 1.39 (1H, m, 6-H$_B$), 1.53 (1H, m, 7-H$_A$), 1.36 (1H, m, 7-H$_B$), 1.71 (1H, tr, *J*= 11.3 Hz, 9-H), 1.96 (1H, m, 11-H), 5.49 (1H, m, 12-H), 1.22 (1H, m, 15-H$_A$), 2.19 (1H, m, 15-H$_B$), 2.12 (1H, dt, *J*= 5.3, 11.4 Hz, 16-H$_A$), 1.96 (1H, m, 16-H$_B$), 3.30 (1H, dd, *J*= 11.2 Hz, 18-H), 1.83 (1H, m, 19-H$_A$), 1.32 (1H, m, 19-H$_B$), 1.46 (1H, m, 21-H$_A$), 1.23 (1H, m, 21-H$_B$), 1.82 (1H, m, 22-H$_A$), 2.04 (1H, m, 22-H$_B$), 1.24 (3H, s, 23-H), 1.02 (3H, s, 24-H), 0.93 (3H, s, 25-H), 1.04 (3H, s, 26-H), 1.30 (3H, s, 27-H), 0.97 (3H, s, 29-H) , 1.02 (3H, s, 30-H). ^{13}C-NMR (400 MHz, DMSO-d_6): δ 39.3 (C-1), 27.4 (C-2), 78.4 (C-3), 39.3 (C-4), 55.5 (C-5), 19.2 (C-6), 32.1 (C-7), 39.4 (C-8), 47.1 (C-9), 37.6 (C-10), 23.2 (C-11), 122.9 (C-12), 145.2 (C-13), 42.3 (C-14), 28.4 (C-15), 23.9 (C-16), 46.8 (C-17), 42.3 (C-18), 46.8 (C-19), 30.4 (C-20), 33.9 (C-21), 33.5 (C-22), 28.5 (C-23), 47.8 (C-24), 14.6 (C-25), 16.9 (C-26), 25.3 (C-27), 180.1 (C-28), 33.2

(C-29), 23.2 (C-30).

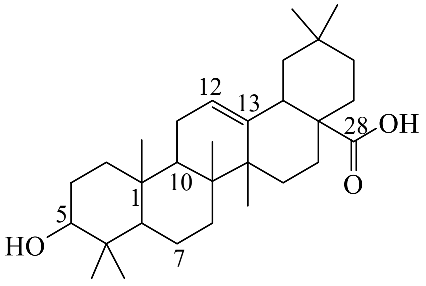

oleanolic acid

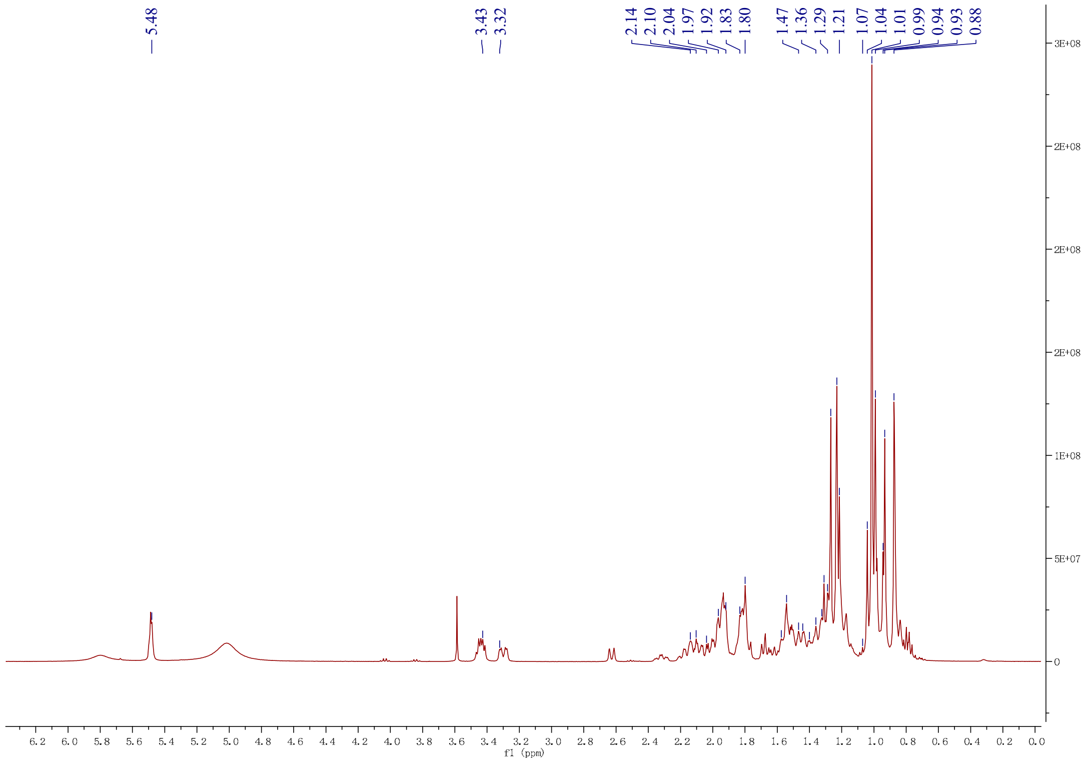

The ^{1}H-NMR spectrum of compound 15

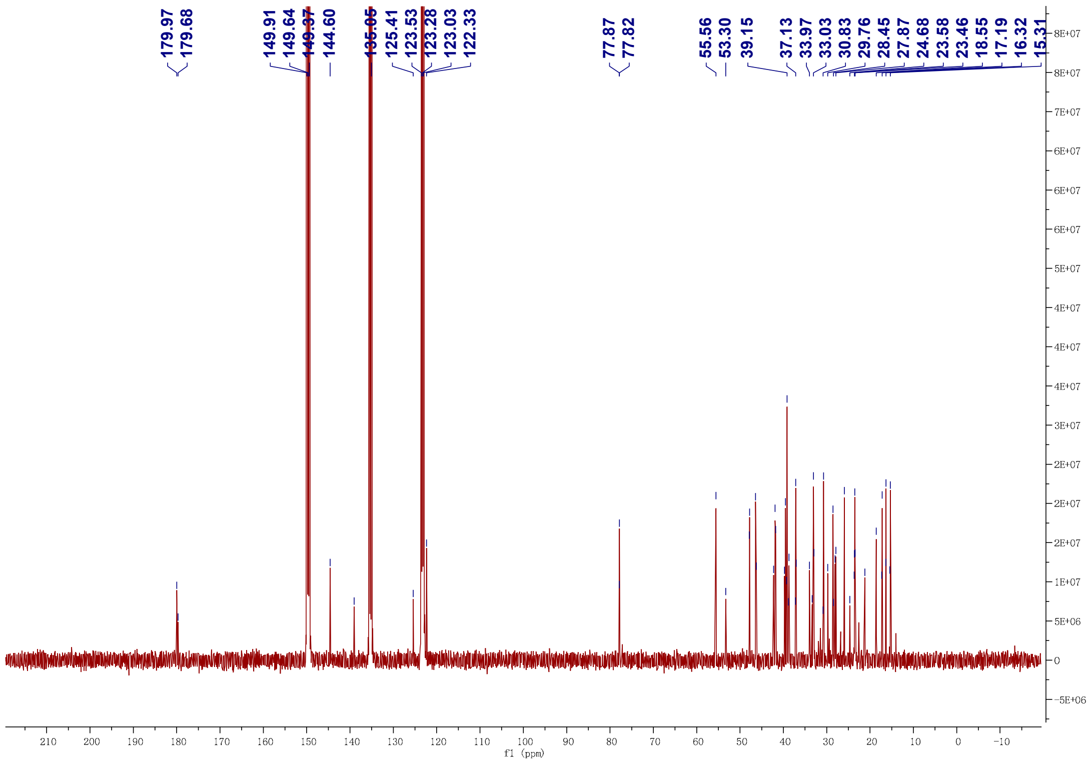

The ^{13}C-NMR spectrum of compound 15

Tyrosol (16): ^{1}H NMR (400 MHz, methanol-d$_4$), δ_H 7.31 (d , *J*=8.3 Hz, H-4,8), 7.16 (d, *J*=8.4 Hz , H-5,7), 4.08 (t , *J*=7.0 Hz, H-1), 3.02 (t, *J*=7.0 Hz, H-2), ^{13}C-NMR (100MHz, methanol-d$_4$): δ64.0(C-1), 39.2(C-2), 130.6(C-3), 130.7(C-4), 116.0(C-5), 156.5(C-6), 116.0(C-7), 130.8(C-8).

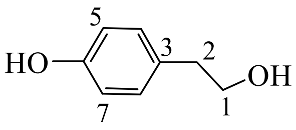

Tyrosol

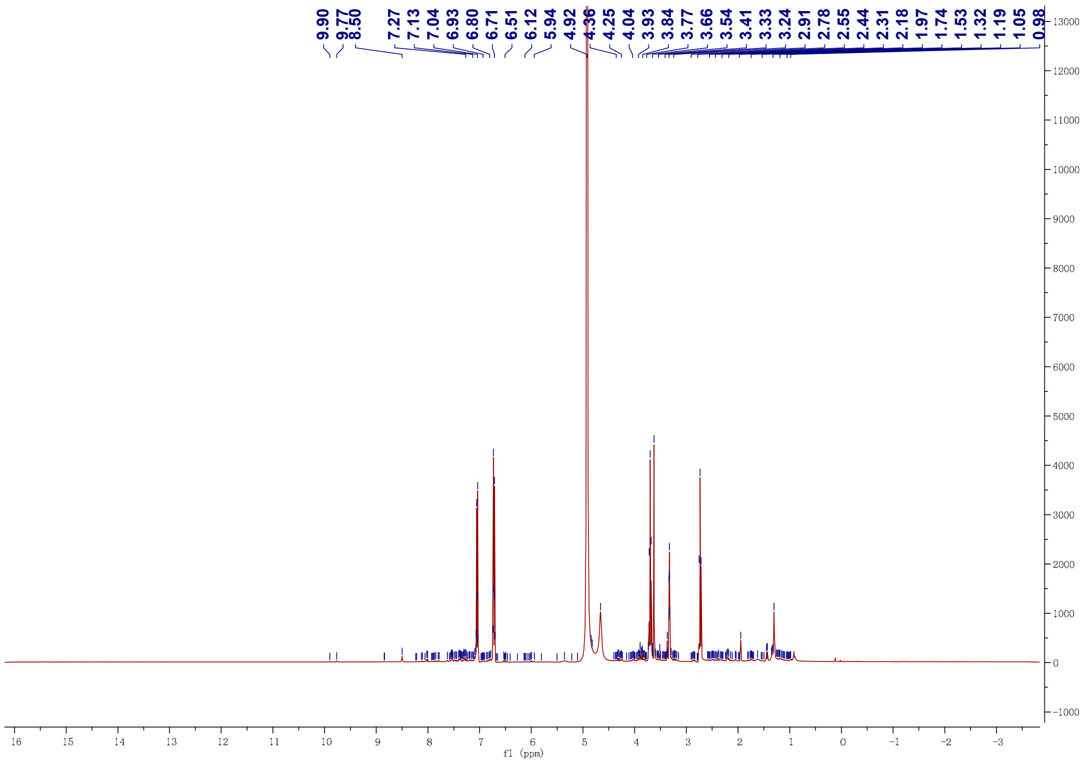

The ^{1}H-NMR spectrum of compound 16

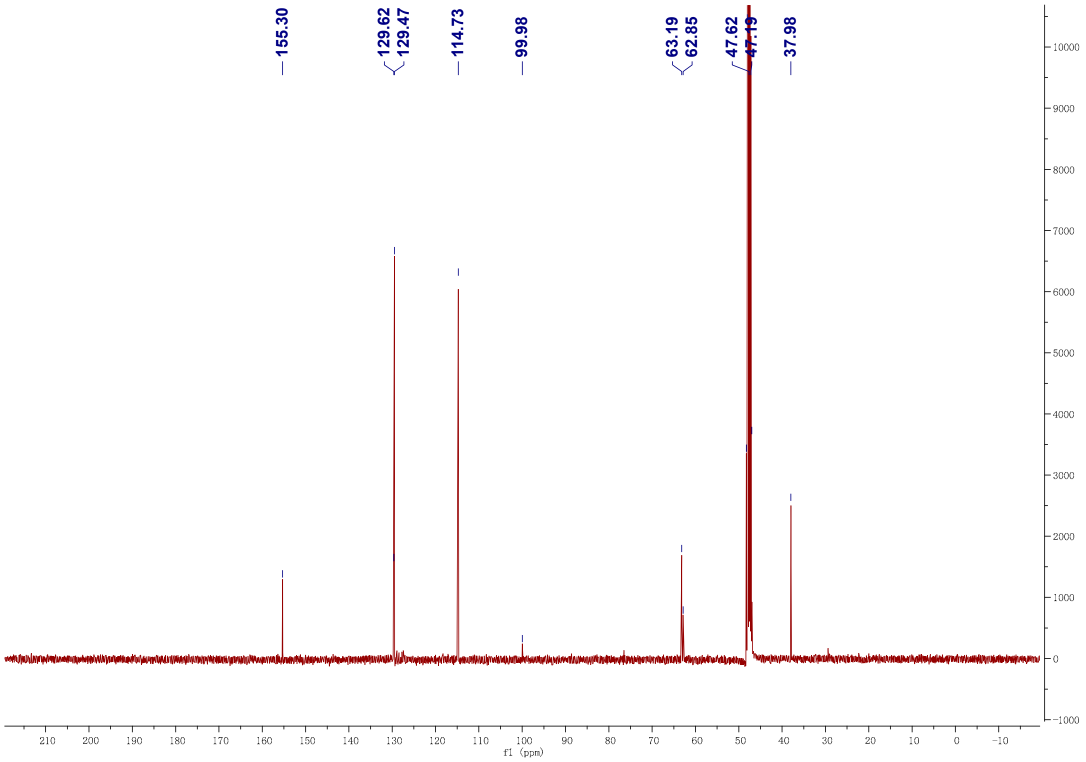

The ^{13}C-NMR spectrum of compound 16
